# GBS hyaluronidase mediates immune suppression in a TLR2/4- and IL-10-dependent manner during pregnancy-associated infection

**DOI:** 10.1128/mbio.02049-23

**Published:** 2023-09-25

**Authors:** Michelle Coleman, Austyn Orvis, Alyssa Brokaw, Anna Furuta, Kavita Sharma, Phoenicia Quach, Avneet Bhullar, Rhea Sanghavi, Shayla Nguyen, Erin Sweeney, Ravin Seepersaud, Blair Armistead, Kristina M. Adams Waldorf, Lakshmi Rajagopal

**Affiliations:** 1 Center for Global Infectious Disease Research, Seattle Children’s Research Institute, Seattle, Washington, USA; 2 Department of Global Health, University of Washington, Seattle, Washington, USA; 3 Department of Obstetrics and Gynecology, University of Washington, Seattle, Washington, USA; 4 Department of Pediatrics, University of Washington, Seattle, Washington, USA; Duke University School of Medicine, Durham, North Carolina, USA

**Keywords:** *Streptococcus agalactiaee*, pregnancy, hyaluronidase, immune suppression, IL-10, HylB

## Abstract

Bacterial infections remain a significant cause of adverse pregnancy outcomes. Ascending infection of group B streptococcus (GBS) or *Streptococcus agalactiae* from the lower genital tract to the amniotic cavity leads to fetal injury, preterm births, or stillbirths. Factors increasing the invasive potential of bacteria at the maternal-fetal interface are poorly understood. Previous studies have indicated that the GBS hyaluronidase (HylB) can enhance systemic infection by breaking down host hyaluronan into disaccharides that dampen protective TLR2 and TLR4 signaling. Here, we examined the importance of hyaluronan receptors such as TLR2, TLR4, and CD44 in defense against GBS infections during pregnancy. While HylB promoted ascending GBS infection in wild-type (WT) and CD44-deficient mice, surprisingly, mice lacking both TLR2 and TLR4 (TLR2/4) were able to curtail these infections. Interleukin-10 (IL-10) and IL-10-expressing macrophages were significantly increased in the uterine tissues of WT mice during infection with HylB-proficient GBS compared with those of TLR2/4-deficient mice, and this likely promotes immune suppression and GBS dissemination. Consistent with these observations, pregnant IL-10-deficient mice exhibited diminished GBS ascension and dissemination. Similarly, the administration of a blocking antibody against the IL-10 receptor (IL-10R) in WT mice diminished ascending GBS infection. Collectively, these observations indicate that HylB promotes immune suppression in a TLR2/4- and IL-10-dependent manner to enhance the invasive potential of GBS during pregnancy-associated infections.

**IMPORTANCE:**

Bacteria such as GBS can cause infections during pregnancy leading to preterm births, stillbirths, and neonatal infections. The interaction between host and bacterial factors during infections in the placenta is not fully understood. GBS secretes a hyaluronidase enzyme that is thought to digest host hyaluronan into immunosuppressive disaccharides that dampen TLR2/4 signaling, leading to increased bacterial dissemination and adverse outcomes. In this study, we show that GBS HylB mediates immune suppression and promotes bacterial infection during pregnancy that requires TLR2, TLR4, and IL-10. Understanding the interaction between host and bacterial factors can inform future therapeutic strategies to mitigate GBS infections.

## INTRODUCTION

Group B streptococci (GBS) are an important cause of infections in humans (1, 2). While GBS commonly reside as a commensal organism in the rectovaginal tracts of healthy adult women, these bacteria can ascend into the pregnant uterus, traffic across the placenta, and lead to either invasive infections of the fetus, or stillbirths, or preterm births (3, 4). Acquisition of GBS through exposure to vaginal fluids during labor and delivery can also lead to infections in human newborns that typically manifest as pneumonia, sepsis, and meningitis (1, 3). The factors that enhance the invasive potential of GBS during perinatal infections are incompletely understood.

A better understanding of the role of host factors during bacterial disease is critical for the development of preventive therapies. One such factor is a major constituent of the host extracellular matrix known as hyaluronan (HA). HA is a high-molecular weight linear, glucosaminoglycan polymer composed of repeating units of the disaccharide (D-glucuronic acid β1–3-N-acetyl-D-glucosamine β1–4) and aids in cell migration and responses to infection and injury (5). Various receptors for hyaluronan have been identified including Toll-like receptor 2 (TLR2) and Toll-like receptor 4 (TLR4) (6 – 9). Recent studies have also revealed the formation of TLR2 and TLR4 heterodimers in response to certain atypical bacterial lipopolysaccharides, such as those produced by the opportunistic pathogen *Ochromobactrum intermedium* (10). During infection or injury, degradation of high-molecular weight HA (HMW-HA) to low-molecular weight HA (LMW-HA) by host hyaluronidases and reactive oxygen species (ROS) is thought to stimulate proinflammatory responses mediated by TLR2 and TLR4 signaling (11, 12). CD44, a cell surface glycoprotein, is another HA receptor, and interactions between these molecules are thought to be important for leukocyte homing and recruitment (13 – 17).

Apart from host hyaluronidases, hyaluronidases are also produced and secreted by bacteria such as group B streptococcus (GBS). GBS hyaluronidase known as HylB (encoded by the *hylB* gene) was described to degrade host hyaluronan into HA disaccharides (18 – 21). Recently, GBS HylB-generated HA disaccharides were shown to block TLR2 and TLR4 signaling in macrophages *in vitro*, likely leading to exacerbated systemic infections (22). We described that clinical GBS strains isolated from invasive disease had higher levels of HylB activity compared with commensal strains (23) and that strains with increased hyaluronidase activity exhibit increased virulence in systemic and pregnancy models of infection (23 – 25). Here, we sought to determine how host hyaluronan receptors contribute to the severity of GBS infections. Interestingly, we observed that mice deficient for both TLR2 and TLR4 (hereafter referred to as TLR2/4) were able to curtail HylB-mediated ascending GBS infections unlike wild-type (WT) or CD44-deficient mice. Notably, we observed that IL-10 and IL-10-expressing macrophages were significantly increased in the uterine tissues of WT mice compared with TLR2/4-deficient mice, during infection with HylB-proficient GBS *in vivo*. Uterine macrophages also produced IL-10 upon GBS infection *in vitro*. These data indicate that GBS-induced IL-10 contributes to a local anti-inflammatory environment in the uterus that facilitates ascending infection. These findings were confirmed using IL-10-deficient mice and by administration of an anti-IL-10 receptor (anti- IL-10R) antibody to WT mice during GBS infection. Both these approaches indicate that TLR2, TLR4, and IL-10 play a critical role in HylB-mediated immune suppression during invasive GBS infections leading to vertical transmission.

## RESULTS

### Hyaluronidase-proficient GBS exhibit decreased ascending infection in pregnant TLR2/4-deficient mice

**Fig 1:**
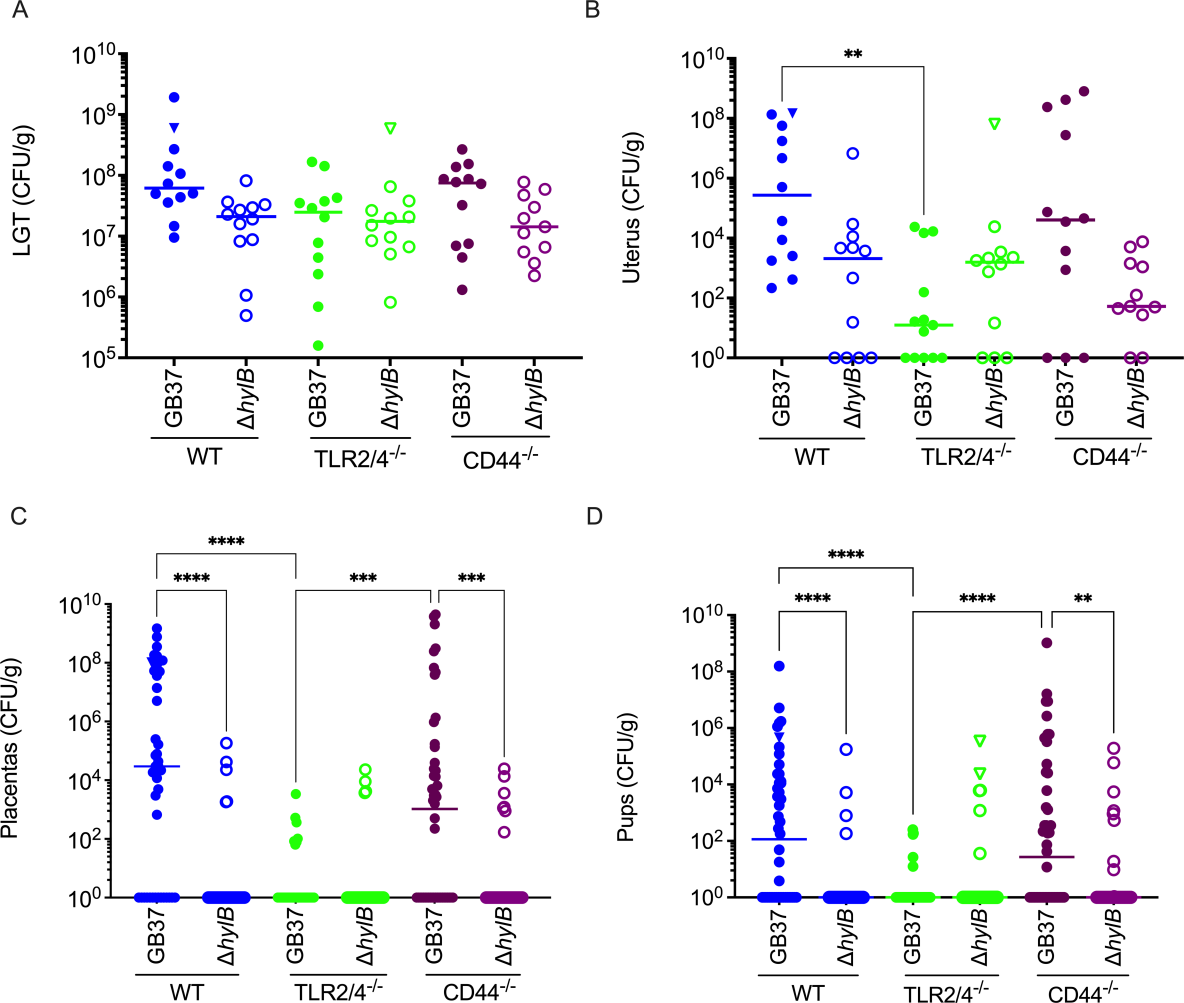
Hyaluronidase-proficient GBS exhibit decreased ascending infection in TLR2/4-deficient mice. Pregnant WT (C57BL/6J) or isogenic TLR2/4-deficient (TLR2/4^−/−^) or CD44-deficient (CD44^−/−^) mice were vaginally inoculated with approximately 1 × 10^8^ CFU of hyaluronidase-proficient WT GBS (strain GB37) or isogenic hyaluronidase-deficient strain GB37Δ*hylB* (*n* = 11–12/group). Tissues of the lower genital tract (LGT), uterus, placentas, and pups were homogenized, and the bacterial burden was enumerated by serial dilution and plating. Individual data with the median are shown for each tissue. Triangle symbols indicate mice that exhibited preterm labor. Kruskal-Wallis test with Dunn’s multiple comparison test was used to assess statistical significance between groups, and significant differences are shown (**P < 0.01, ***P < 0.001, and ****P < 0.0001).

We previously showed that increased expression of hyaluronidase confers hypervirulence to a non-hemolytic GBS strain (GB37) (24) and promotes ascending infection and preterm labor (23, 25). As TLR2 and TLR4 are receptors for GBS hyaluronidase-generated disaccharides (22), we hypothesized that ascending GBS infection in pregnant TLR2/4-deficient mice may be different from WT mice, due to the absence of TLR2/4-mediated immune signaling as observed in TLR2-deficient mice during systemic infection (22). To test this hypothesis, pregnant WT (C57BL6) and TLR2/4-deficient mice at day 15 of gestation were vaginally inoculated with WT GBS (strain GB37) or the isogenic hyaluronidase-deficient strain GB37∆*hylB* (*n* = 11–12/group, ~1 × 10^8^ CFU) using methods described previously (23). As CD44 is a HA receptor that has been described as integral for leukocyte homing and recruitment (15 – 17), CD44-deficient mice were also included in these studies. The GBS-infected mice were then monitored for signs of preterm labor/birth (i.e., vaginal bleeding and pups in cage). Upon signs of preterm labor or at the experimental end (3 days post-inoculation), mice were euthanized and tissues of the lower genital tract (LGT), uterus, entire pups, and placenta were harvested, homogenized, serially diluted, and plated to enumerate GBS CFU. The results shown in Fig. 1 indicate that GBS vaginal colonization was not significantly different between WT and isogenic TLR2/4- or CD44-deficient mice inoculated with either GB37 or GB37Δ*hylB* (see LGT [lower genital tract], Fig. 1A). As observed previously (23), bacterial burden in the uterus, placenta, and fetal tissues was higher for GB37 compared with GB37∆*hylB* in WT mice (Fig. 1B through D). Interestingly, TLR2/4-deficient mice inoculated with GB37 exhibited significantly reduced bacterial infection in the uterus, placenta, and fetal tissues when compared with WT mice (Fig. 1B through D). In contrast, GBS dissemination in CD44-deficient mice was not significantly different from that in WT mice for GB37 or GB37∆*hylB* strains (Fig. 1A through D). Additionally, GBS dissemination in the uterine, placenta, and fetal tissues of single TLR2 or TLR4 knockout mice was not significantly different from that of WT mice (see Fig. S1B through D ). We also did not recover any bacteria from PBS-inoculated pregnant WT and TLR2/4-deficient mice (Fig. S2). Collectively, these results indicated that ascending infection of hyaluronidase-proficient GB37 was significantly diminished in TLR2/4-deficient mice when compared with WT mice, suggesting that HylB-mediated *in utero* transmission relies on TLR2/4 signaling.

### IL-10-expressing macrophages were more abundant in the uterine tissues of WT mice during HylB-mediated ascending GBS infection

**Fig 2:**
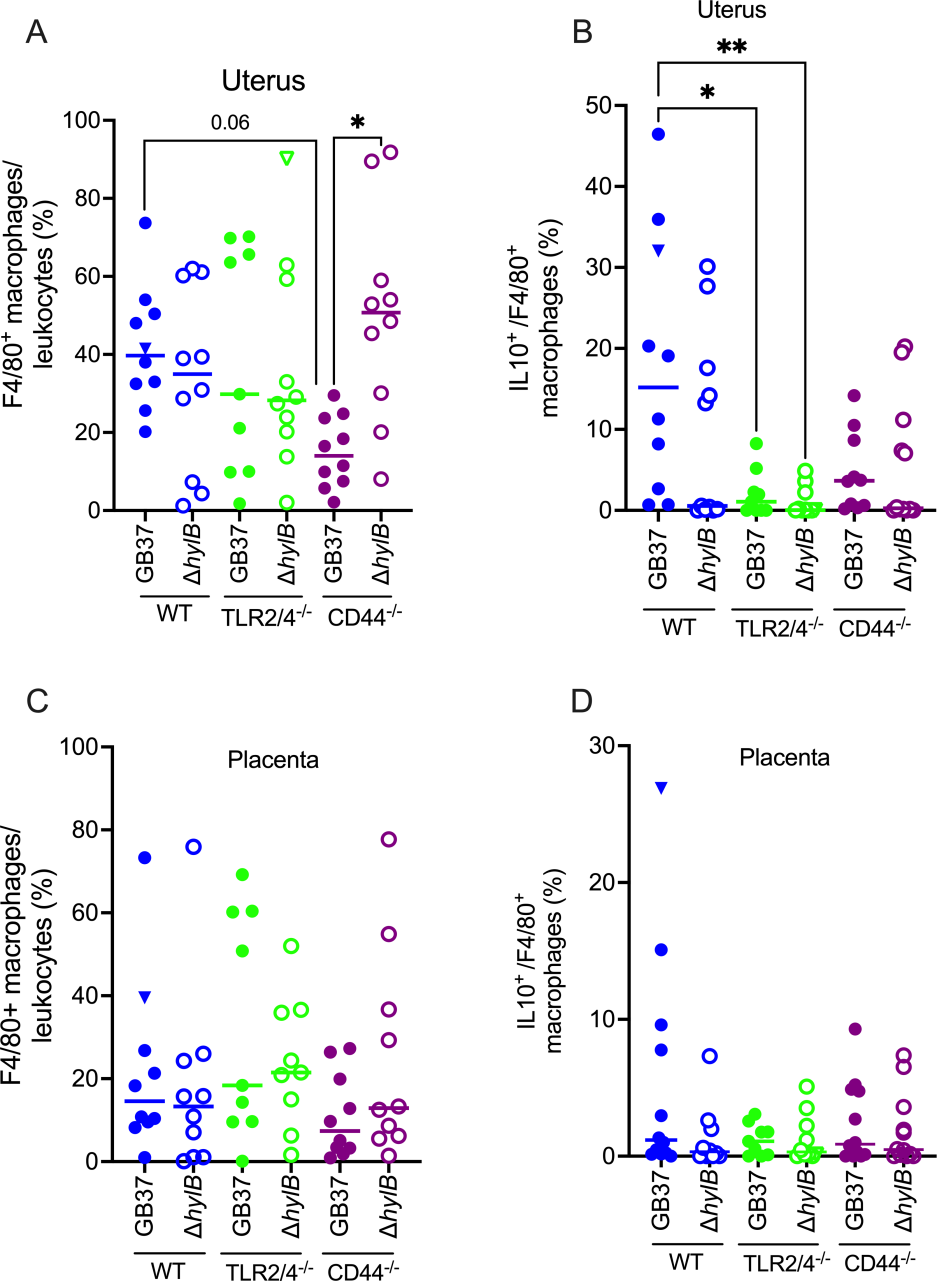
Increased IL-10-expressing macrophages in uterine tissues of WT mice infected with hyaluronidase-proficient GBS. Flow cytometry was used to determine macrophage frequencies in uterine and placental samples of WT , TLR2/4- and CD44-deficient mice that were vaginally inoculated with GB37 or GB37Δ*hylB* (*n* = 9–10/group). Frequencies of F4/80^+^ macrophages and F4/80^+^ IL10^+^ macrophages are shown as percentages of the indicated population for each tissue type. Triangle symbols indicate mice that exhibited preterm labor. Kruskal-Wallis test with Dunn’s multiple comparison test was used to assess statistical significance between groups and significant differences are shown (*P < 0.05, **P < 0.01).

To understand why ascending GBS infection was lower in TLR2/4-deficient mice compared with WT mice, we evaluated the nature of immune cells that were recruited to the uterus and placenta during infection. Thus, cells were isolated from the uterus and placentas of WT and TLR2/4- and CD44-deficient mice infected with GB37 or GB37∆*hylB* and flow cytometry was used to determine frequencies of leukocytes including neutrophils (Gr1^+^), macrophages (F4/80^+^), and IL-10-expressing macrophages (see gating strategy in Fig. S3). The results shown in Fig. 2A indicate that macrophages (F4/80^+^) were present in uterine tissues of WT and TLR2/4- and CD44-deficient mice infected with GBS; however, frequencies of uterine macrophages were lower in CD44-deficient mice infected with GB37 compared with isogenic mice infected with GB37∆*hylB* or when compared with GB37-infected WT mice (see Fig. S4 for representative dot plots). Interestingly, the frequencies of F4/80^+^IL-10^+^ macrophages were significantly greater in WT mice compared with TLR2/4-deficient mice that were infected with GB37 (Fig. 2B; Fig. S5). This increase in anti-inflammatory IL-10-producing macrophages likely contributes to immune suppression, facilitating heightened GBS dissemination as observed in WT mice compared with the TLR2/4-deficient mice. Flow cytometric analysis of macrophage frequencies in placental samples of these mice was not statistically different (Fig. 2C and D; Fig. S6 and S7). Unlike macrophages, Gr1^+^ neutrophil frequencies were not statistically significant between groups for either tissue (Fig. S8). Macrophages and neutrophils were also not significantly different in uterine and placental tissues of pregnant WT and TLR2/4-deficient mice that were mock infected with PBS (Fig. S9). We further confirmed that the TLR2/4-deficient mice were not inherently deficient in IL-10 production as TLR2/4-deficient macrophages released IL-10 upon exposure to CpG (a TLR9 agonist) that was not significantly different from that of CpG-treated WT macrophages (Fig. S10). Collectively, these results suggest that HylB-mediated immune suppression of TLR2/4 is associated with increased rates of IL-10^+^ macrophages in uterine tissues, which promotes ascending infection.

### Higher IL-10 and lower MIP-1α, MIP-2α, and IL-1β levels in uterine tissues of GB37-infected WT mice compared with TLR2/4-deficient mice

**Fig 3:**
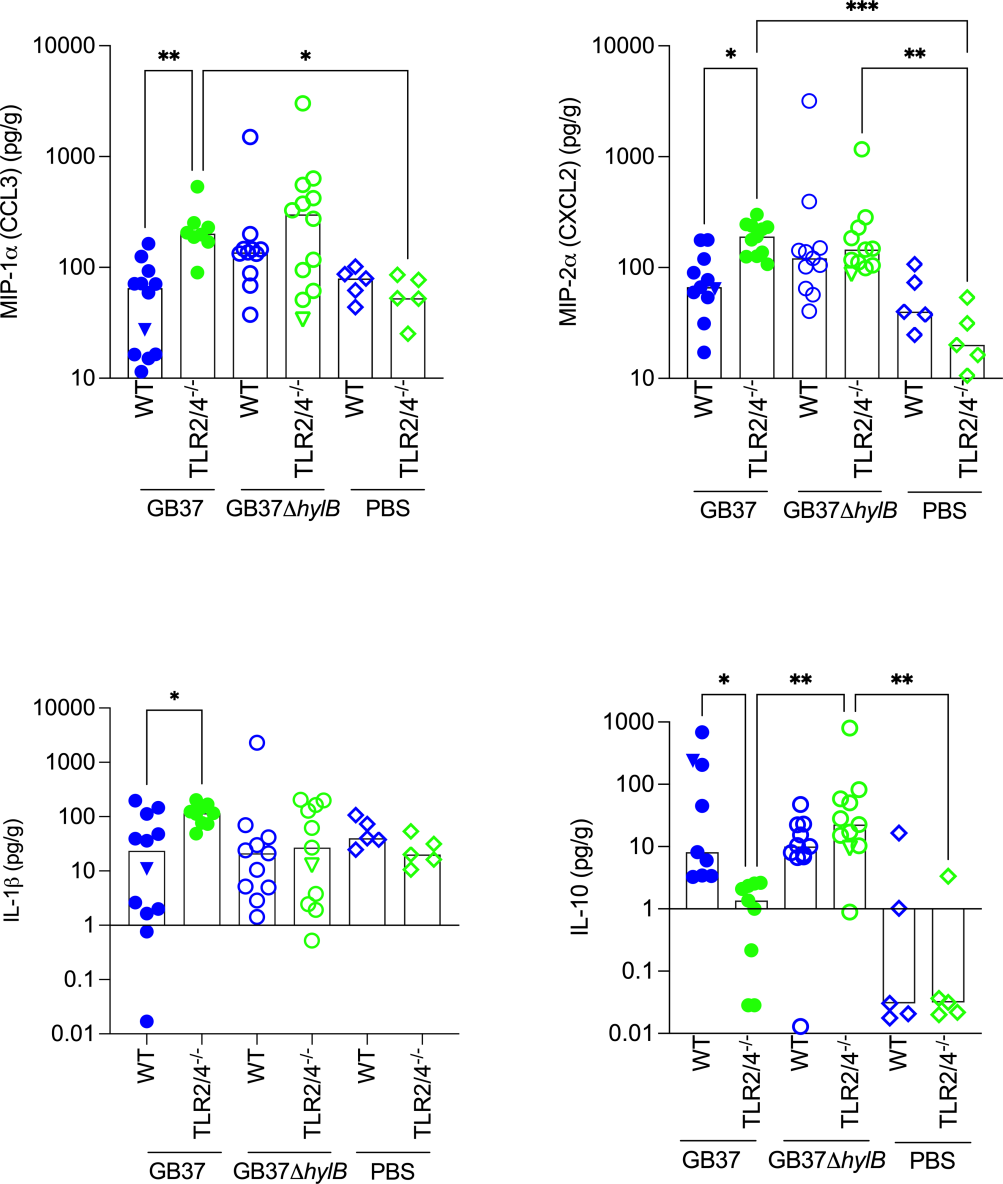
Increased MIP-1α, MIP-2α, and IL-1β and lower IL-10 in GB37-infected uterine tissues of TLR2/4-deficient mice. Cytokine and chemokine concentrations in uterine tissue lysates from WT and TLR2/4-deficient mice following vaginal infection with GB37 or GB37Δ*hylB* (*n* = 9–11/group) or PBS (*n* = 5/group) were determined using multiplex assays. MIP-1α, MIP-2α, and IL-1β were significantly increased in GB37-infected uterine tissues of TLR2/4-deficient mice compared with WT mice. Conversely, IL-10 levels were significantly lower in uterine tissues of TLR2/4-deficient mice when compared with WT mice infected with GB37 or TLR2/4-deficient mice infected with GB37Δ*hylB*. Kruskal-Wallis test with Dunn’s multiple comparison test was used to assess statistical significance between groups, and significant differences are shown (*P < 0.05, **P < 0.01, and ***P < 0.001).

We also performed Luminex assays to compare levels of inflammatory cytokines in the uterine tissues of pregnant WT and TLR2/4-deficient mice that were vaginally inoculated with WT GB37 or GB37Δ*hylB*. The results shown in Fig. 3 indicate that the inflammatory mediators MIP-1α, MIP-2α, and IL-1β were significantly higher in uterine tissues of TLR2/4-deficient mice compared with WT mice infected with GB37. Interestingly, IL-10 levels were significantly lower in uterine tissues of TLR2/4-deficient mice compared with WT mice that were infected with GB37, or TLR2/4-deficient mice infected with GB37Δ*hylB*. Taken together, these data suggest that increased expression of proinflammatory cytokines along with decreased levels of IL-10 and IL-10-expressing macrophages in GB37-infected TLR2/4-deficient mice contributes to diminished ascending infection.

### Uterine macrophages from WT mice exhibit increased IL-10 expression when exposed to GB37

**Fig 4:**
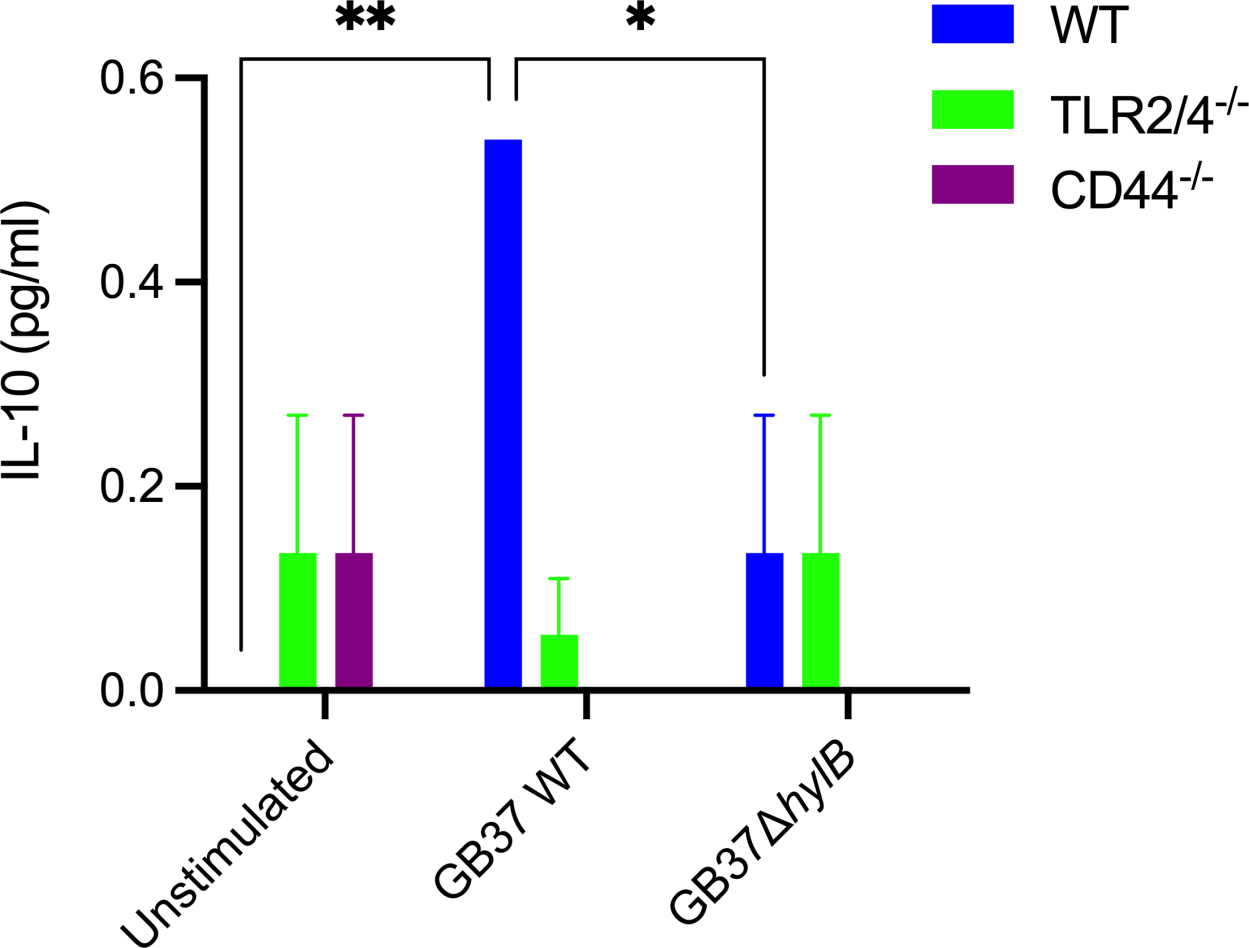
Increased IL-10 production in GB37-infected uterine macrophages of WT mice versus TLR2/4-deficient mice. F4/80^+^ uterine macrophages isolated from naïve (uninfected) pregnant WT or TLR2/4-deficient mice were infected with GB37 or GB37Δ*hylB* at MOI 1 or mock infected with PBS for 4 h, after which IL-10 concentrations in supernatant were quantified via Luminex. Statistical significance was assessed using a two-way ANOVA with Tukey’s multiple comparison test (*P < 0.05, **P < 0.01).

We next assessed if GB37 stimulated IL-10 expression in uterine macrophages *in vitro*. To this end, F4/80^+^ macrophages were isolated from uterine tissues of naïve (uninfected) pregnant WT or TLR2/4-deficient mice at embryonic days E13−14. The uterine macrophages were subsequently infected with WT GB37 or GB37Δ*hylB* at a multiplicity of infection (MOI) of 1 or treated with control PBS for 4 h, after which IL-10 concentrations in the supernatant were quantified via Luminex. The results shown in Fig. 4 indicate that upon exposure to GB37, uterine macrophages isolated from WT mice exhibited significantly increased IL-10 production when compared with those from TLR2/4-deficient mice. This increase in IL-10 was not observed with hyaluronidase-deficient GBSΔ*hylB*. These data further confirm that hyaluronidase-proficient GBS stimulates IL-10 production in uterine macrophages of WT mice via TLRs 2 and 4.

### Cytokine expression in GB37-infected uterine macrophages is dampened by IL-10

**Fig 5:**
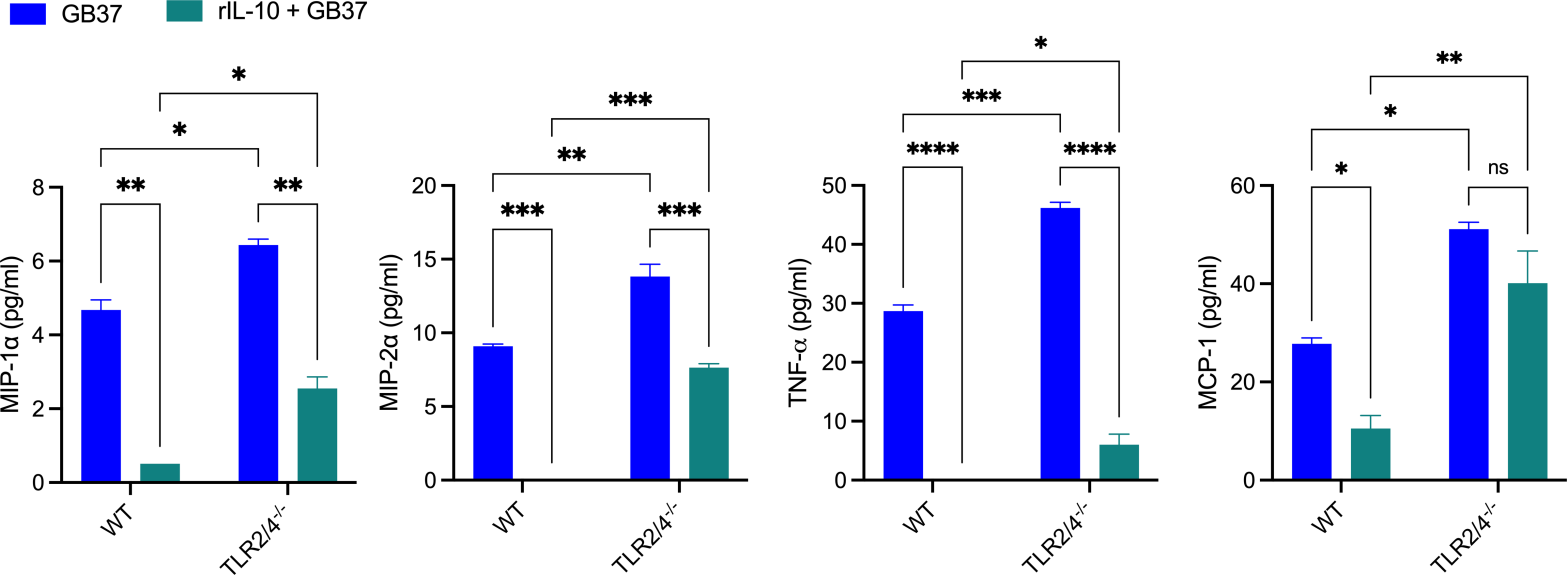
Cytokine expression in GB37-infected uterine macrophages is dampened by IL-10. F4/80^+^ macrophages were isolated from naïve (uninfected) uterine tissues of pregnant WT or TLR2/4-deficient mice at embryonic days 13–14 and pre-treated with either rIL-10 (100 pg/mL) or PBS for 1 h prior to infection with GB37 at MOI 1. After 4 h infection, cytokine concentrations in the supernatant were quantified via Luminex. Statistical significance was assessed using a two-way ANOVA with Tukey’s multiple comparison test (*P < 0.05, **P < 0.01, ***P < 0.001, and ****P < 0.0001).

We examined the effect of IL-10 on the cytokine production of GB37-infected uterine macrophages *in vitro*. For this, F4/80^+^ macrophages were isolated from uterine tissues of pregnant WT or TLR2/4-deficient mice on embryonic days E13–14 as indicated above. The macrophages were pre-treated for an hour with 100 pg/mL rIL-10 (550070, BD) or control PBS prior to infection with WT GB37 at MOI 1 for 4 h, after which cytokine and chemokine concentrations in the supernatant were quantified via Luminex. The results shown in Fig. 5 indicate that TLR2/4-deficient macrophages infected with GB37 exhibit significantly increased concentrations of MIP1-α, MIP2-α, TNF-α, and MCP-1 when compared with WT. Furthermore, pre-treatment with recombinant IL-10 significantly decreased the production of the above cytokines and chemokines in both WT and TLR2/4-deficient macrophages. These data confirm that IL-10 dampens proinflammatory responses to GBS in uterine macrophages.

### IL-10-deficient mice exhibit decreased ascending GBS infection

**Fig 6:**
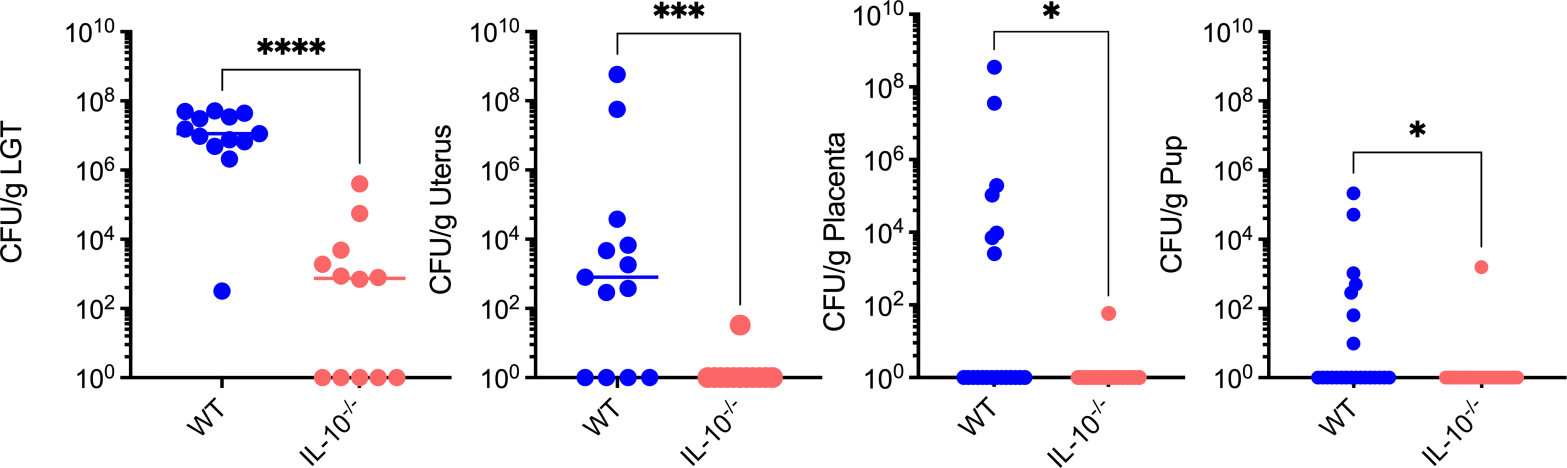
Pregnant IL-10-deficient mice exhibit diminished GBS ascension and dissemination. Pregnant WT or isogenic IL-10-deficient mice (IL-10^−/−^) at embryonic day E15 were vaginally inoculated with approximately 1 × 10^8^ CFU of hyaluronidase-proficient WT GB37 (*n* = 11–12/group). Mice were monitored for signs of preterm labor up to 3 days post-infection. Then, tissues of the LGT, uterus, placentas, and pups were homogenized and the bacterial burden was enumerated by serial dilution and plating. Individual data with the median/s are shown. Mann-Whitney test was used to assess statistical significance between groups (*P < 0.05, ***P < 0.001, and ****P < 0.0001).

Our results suggest that decreased IL-10 production during GBS infection in TLR2/4-deficient mice reduces the severity of ascending infection and may promote bacterial clearance. To test this possibility, pregnant WT mice and IL-10-deficient mice at E15 were vaginally inoculated with 1 × 10^8^ CFU of WT GB37. Mice were euthanized 72 h post-inoculation. Tissues of LGT, uterus, placentas, and pups were homogenized, and CFU were determined as described earlier. The results shown in Fig. 6 indicate that IL-10-deficient mice exhibited diminished colonization and dissemination of GBS in all reproductive tissues.

### Administration of anti-IL-10R antibody diminished ascending GBS infection

To further confirm these observations, we hypothesized that administration of an IL-10 receptor-neutralizing antibody (anti-IL-10R) could block IL-10 signaling and promote a reduction in the GBS burden. We first confirmed that administration of the anti-IL-10R blocked the IL-10R in mice. To this end, female WT mice received 100 μg of anti-IL-10R mAb, intraperitoneally 0–96 h prior to euthanasia. Control mice received the isotype control NA/LE Rat IgG1 mAb. Single-cell suspensions were then generated from enzymatically digested uterine tissue at various times post-administration. The frequency of IL-10R expression in uterine cells was determined by flow cytometry using a PE-conjugated anti-IL-10R of the same clonality as the antibody administered *in vivo*. The results shown in Fig. S11 indicate that *in vivo* administration of the anti-IL-10R antibody significantly diminished fluorescent staining of IL-10R+ cells in the uterus for up to 48 h.

**Fig 7:**
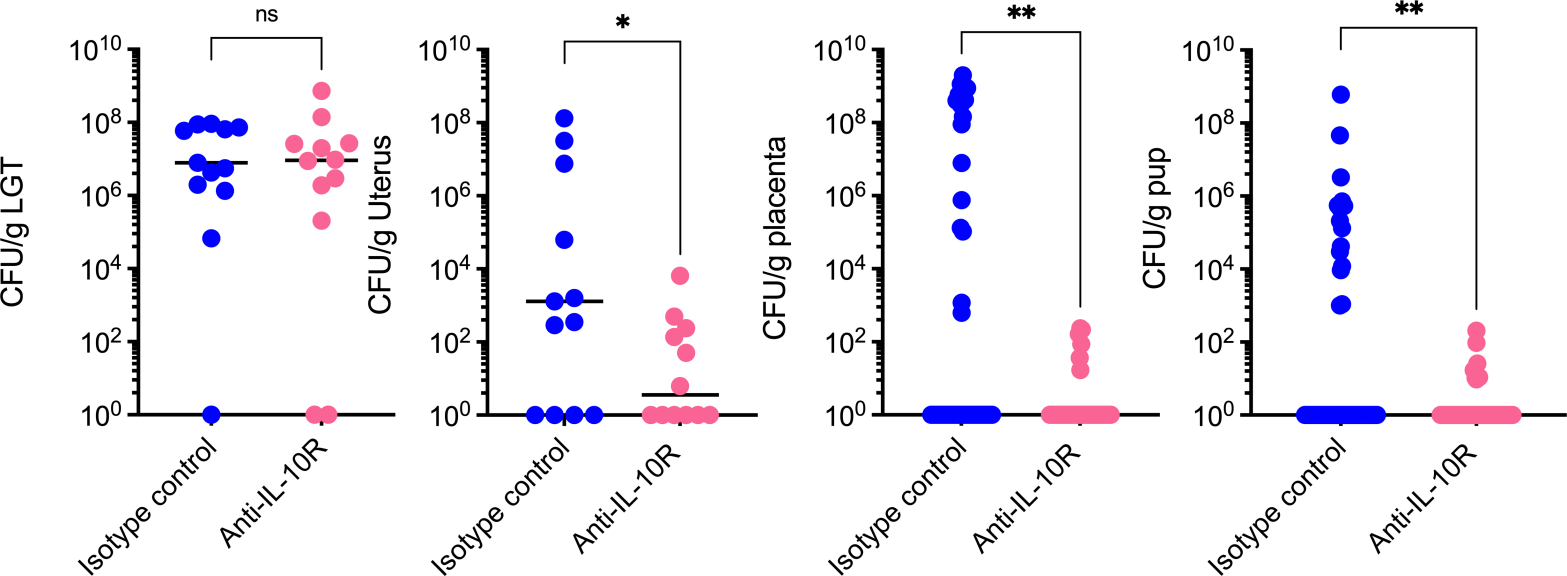
Administration of anti-IL-10 receptor antibody (anti-IL-10R) diminished ascending GBS infection. Pregnant WT mice were injected intraperitoneally with 100 μg of NA/LE anti-IL-10R or isotype control at embryonic days E12–E13, 24 h prior to vaginal inoculation with 1 × 10^8^ CFU of WT GB37 (*n =* 12/group). Mice were monitored for signs of preterm labor up to 3 days post-infection. Then, tissues of the LGT, uterus, placentas, and pups were homogenized, and CFU were determined by serial dilution and normalized to tissue weight. Individual data with the median is shown for each tissue. Mann-Whitney test was used to assess statistical significance between groups (*P < 0.05, **P < 0.01).

We then examined the impact of the administration of the anti-IL10R antibody in ascending GBS infection. Pregnant WT mice (gestational age 12–13 days) were injected intraperitoneally with 100 μg of anti-IL10R mAb or isotype control antibody 24 h prior to vaginal inoculation with 1 × 10^8^ CFU of WT GB37. Mice were euthanized 72 h post-inoculation, and tissues of the LGT, uterus, placentas, and pups were homogenized, and bacterial CFU were determined by serial dilution as described earlier. The results shown in Fig. 7 indicate that mice that received the anti-IL-10R antibody exhibited diminished ascending GBS infection in the uterus, placenta, and pups compared with mice that received the isotype control. Collectively, our results indicate that GBS HylB augments uterine IL-10 production in a TLR2/4-dependent manner, which promotes ascending GBS infection.

## DISCUSSION

**Fig 8:**
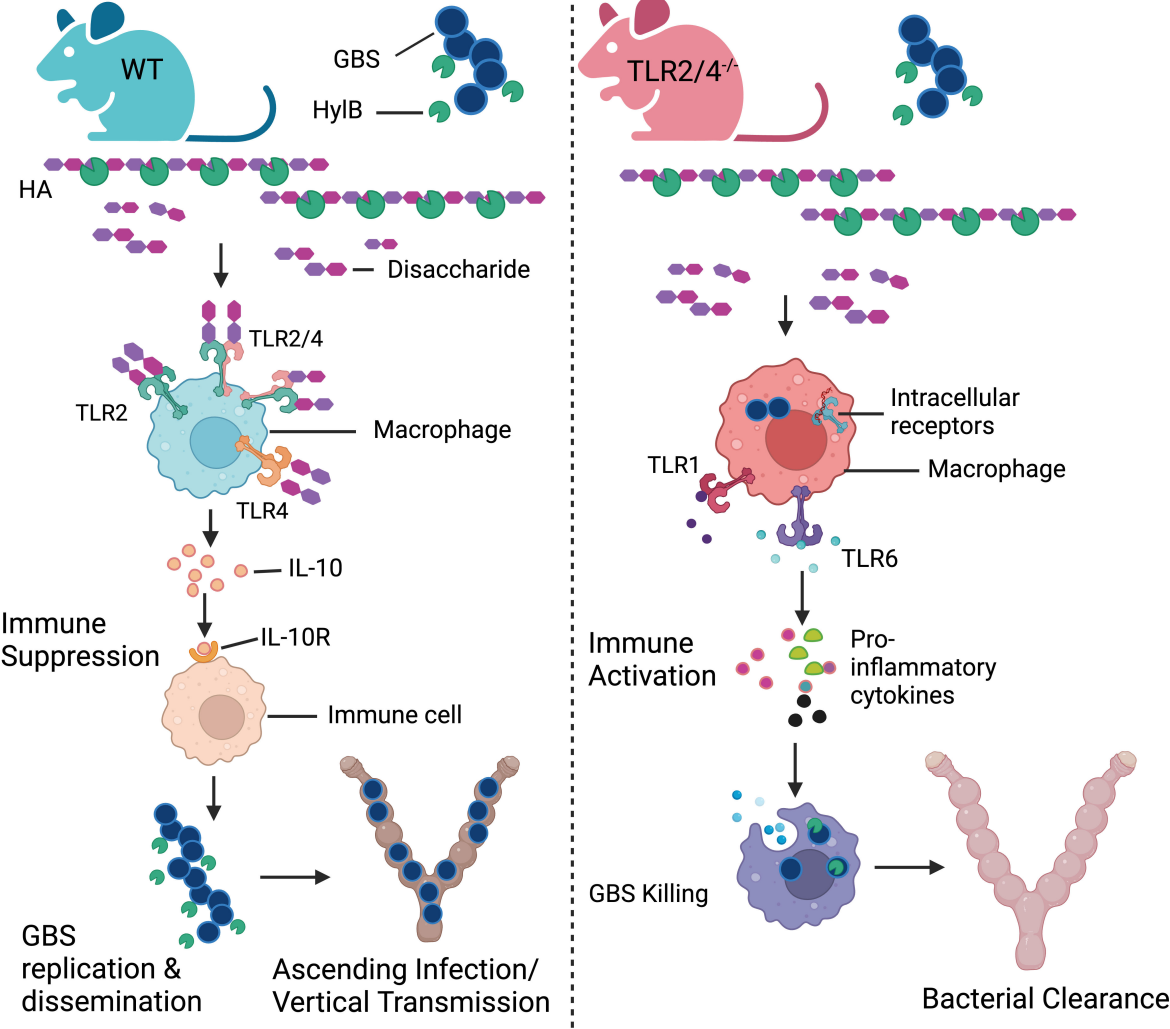
Proposed model of HylB-mediated TLR2/4 suppression during ascending GBS infection. Group B *Streptococcus* (GBS) secretes the hyaluronidase enzyme (HylB), whose activity cleaves hyaluronan (HA) into disaccharides. HA disaccharides bind to TLR2, TLR4, and potentially to TLR2/4 heterodimers (10) on immune cells (e.g., macrophages) promoting immune suppression through IL-10 production, which facilitates GBS dissemination and virulence during pregnancy. In the absence of both TLR2 and TLR4, IL-10 is not induced by HA disaccharides, and the proinflammatory immune responses generated against GBS-derived PAMPs and DAMPs via host receptors (e.g., TLR1, TLR6, and host intracellular receptors) lead to diminished GBS burden and bacterial clearance during pregnancy.

Hyaluronan is a critical extracellular matrix component that is essential for responses to injury and infection. Depletion of HA in the cervix and vagina has been reported to cause anomalies in epithelial cell differentiation leading to increased permeability that promoted *E. coli*-induced preterm births (26). In this study, we describe the impact of host hyaluronan receptors, namely, TLR2, TLR4, and CD44, on the progression of invasive, ascending GBS infections. Previous studies have shown that the GBS hyaluronidase (HylB) breaks down host hyaluronan into disaccharides, which suppress TLR2 and TLR4 signaling to diminish macrophage cytokine responses (22) and neutrophil ROS responses (25). Consequently, this immune suppression is thought to facilitate increased virulence of HylB-proficient GBS strains. Surprisingly, in this study, we observed that pregnant mice deficient for both TLR2 and TLR4 (TLR2/4) were able to curtail the ascending infection of a hyaluronidase-proficient WT GBS strain. Increased IL-10-expressing macrophages and IL-10 levels observed in the uterine tissues of GBS-infected WT mice compared with TLR2/4-deficient mice may explain the increased bacterial burden observed in these mice. In the absence of TLR2 and TLR4, the HA disaccharides could not induce an IL-10-mediated immune suppression resulting in a strong proinflammatory innate immune response to other PAMPs (pathogen-associated molecular patterns) and/or DAMPs (damage-associated molecular patterns) that enabled control of the pathogen (Fig. 8). This hypothesis is supported by our observations that there was a significant increase in the proinflammatory mediators MIP-1α, MIP-2α, and IL-1β in GBS-infected uterine tissues of TLR2/4-deficient mice versus WT mice. Furthermore, when uterine macrophages were pre-treated with recombinant IL-10 prior to GBS infection, the production of MIP-1α, MIP-2α, TNF-α, and MCP-1 was significantly diminished from both WT and TLR2/4-deficient cells. Together, these results support an immunosuppressive and deleterious role of IL-10 on host control of ascending GBS infection.

The importance of uterine responses in TLR2/4-mediated immune suppression due to GBS HylB is consistent with our prior observations (23). Uterine tissues express high levels of TLRs including TLR1, TLR2, TLR4, and TLR6 (27). In mice, uterine TLR2 and TLR4 expression increases during gestation, whereas placental TLR2 and TLR4 expression decreases as gestation progresses (28). The combined absence of both TLR2 and TLR4 resulted in significant resistance to ascending GBS infections, in contrast with mice lacking either TLR2 or TLR4 alone (Fig. 1; Fig. S1); this was unsurprising as these two receptors have been described to be compensatory during acute bacterial infections (29). Our results further revealed that macrophage recruitment was significantly lower in the uterine tissues of CD44-deficient mice infected with the hyaluronidase-proficient GB37 compared with either WT mice or isogenic mice infected with hyaluronidase-deficient GBS. These observations suggest that the hyaluronan receptor CD44 is important for macrophage recruitment during infection with hyaluronidase-proficient GBS. Thus, CD44 is necessary for HA disaccharides to recruit macrophages, whereas cytokine and chemokine expression is modulated by HA disaccharide binding to TLR2 and TLR4. Collectively, these data emphasize the importance of immune signaling mediated by uterine hyaluronan receptors during ascending GBS infection.

IL-10 is an anti-inflammatory cytokine that has been associated with innate immune suppression during bacterial infections including GBS (30 – 32). For example, susceptibility of adult and neonatal mice to GBS infection was suggested to be dependent on early host IL-10 production (31, 32). These studies found that glyceraldehyde-3-phosphate dehydrogenase (GAPDH) acts as an extracellular virulence factor that rapidly induces IL-10 production and exacerbates GBS virulence in mice (31). Additionally, inhibition of IL-10 by maternal recombinant GAPDH antibodies promoted immunity in newborn mice against GBS infections (32). Notably, TLR2-dependent IL-10 production was shown to impair immune cell recruitment to infected tissues during GBS neonatal sepsis (33) and neonatal mice deficient in TLR2 exhibited reduced levels of IL-10 upon GBS infection that were ultimately associated with bacterial clearance (33).

A role for IL-10 in the inhibition of proinflammatory immune responses and diminished bacterial burden has also been reported for other pathogens. For example, IL-10 inhibits the production of MIP-1α in monocytes and alveolar macrophages in response to lipopolysaccharides (LPS) and IL-1β (34). In a mouse model of *Pseudomonas aeruginosa* infection, overexpression of IL-10 was associated with reduced neutrophil recruitment to the lungs and increased bacterial burden (35). IL-10 has also been associated with the ability of *Mycobacterium tuberculosis* to evade host immune responses, and consequently, blockade of the IL-10 receptor was noted to alleviate these infections (36, 37). Similarly, inhibition of the IL-10 receptor using a mAb was shown to diminish lipopolysaccharide-induced proinflammatory signaling in non-pregnant, cynomolgus macaques (38). In a *Streptococcus pneumoniae* model of infection, IL-10-deficient mice demonstrated elevated bacterial clearance in comparison with WT mice, which was associated with increased TNF-α in bronchoalveolar lavage fluid; however, this was also associated with sustained neutrophil infiltration, lung damage, and increased mortality (39).

During pregnancy, IL-10 is regarded as playing an important anti-inflammatory role in the uterus and placenta during normal pregnancy, labor, and preterm labor. IL-10 concentrations were significantly higher in human amniotic fluid during spontaneous labor at term and preterm than prior to labor indicating a role for IL-10 in modulating labor-associated inflammatory responses (40). In the mouse, decidual and myometrial macrophages release IL-10 and TGF-β, with IL-10 levels increasing as gestation progresses (41). However, there is a paucity of literature addressing the localized production of IL-10 by specific cell types in response to bacterial infections and the consequent impact on the progression of infection. Several studies using LPS as a model of a Gram-negative bacterial infection have indicated that IL-10 administration prevents preterm birth in rodent models (42, 43). In parallel, several therapeutics preventing LPS-associated preterm birth (NF-κB or endothelin-1 receptor blockade) were associated with increased IL-10 production and reduced preterm birth (44, 45). However, the LPS model is limited in understanding the impact of the cytokine milieu during bacterial invasion and whether IL-10 production might enhance the invasive potential of other commensal bacteria.

Suppression of IL-10R signaling during human preterm labor may be beneficial for maternal-placental control of the pathogen but would require careful titration given the beneficial actions of IL-10 in normal pregnancy. IL-10 is a highly pleiotropic cytokine in pregnancy and has key roles in the maintenance of immune tolerance at the maternal-fetal interface (46). The immunosuppressive actions of IL-10 occur through the inhibition of Th1-type responses, in addition to limiting the innate effector functions of macrophages and dendritic cells (47). IL-10 also plays a key role in protecting the placenta’s vasculature and reducing preeclampsia-like disease in rodents (48, 49). The offspring of IL-10^−/−^ mice are also susceptible to uterine NK cell-mediated fetal resorption, intrauterine growth restriction, or preterm birth when exposed to low doses of intraperitoneal LPS (50, 51). Although there is a wealth of evidence supporting the beneficial effect of IL-10 on the maintenance of healthy pregnancies, a negative consequence is the impaired host control of pathogens at the maternal-fetal interface. Studies to investigate the efficacy and safety of inhibiting IL-10 signaling in pregnant women with preterm labor should be tested with the goal of improving host clearance of pathogens and prolonging pregnancy. The pregnancy outcomes of hypertensive disease, intrauterine growth restriction, and stillbirth should be carefully monitored in the context of pre-clinical and Phase 1 studies in pregnant patients.

In summary, we found that blocking IL-10 signaling or deficiencies in IL-10 result in diminished ascending GBS infection during pregnancy. Although ascending GBS infection can in part be attributed to diminished colonization in IL-10-deficient mice (Fig. 6), no significant differences in lower genital tract colonization were noted in mice that received the anti-IL-10R antibody (Fig. 7). Collectively, our results indicate that inhibition of IL-10 signaling reduces GBS burden in reproductive tissues during pregnancy. Whether antagonism of IL-10 can be a viable approach to preventing invasive perinatal GBS infections is unknown, but it represents an interesting therapeutic strategy that might be leveraged to prevent preterm births.

## MATERIALS AND METHODS

### Chemicals and bacterial strains

Chemicals in this study were purchased from Sigma Aldrich unless stated otherwise. The WT GBS strain GB37 used in these studies is a serotype V clinical isolate from an infected human newborn (52, 53). The isogenic hyaluronidase-deficient strain was previously derived (25). GBS cultures were grown in tryptic soy broth (TSB) or on tryptic soy agar (TSA; Difco Laboratories) at 30°C or 37°C in 5% CO_2_ and monitored at 600 nm.

### Murine model of ascending GBS infection

Six- to eight-week-old WT C57BL/6 and isogenic CD44-, IL-10-, TLR2-, and TLR4-deficient mice were obtained from The Jackson Laboratory and bred in-house for ascending infection studies. TLR2/4-deficient mice (54) was obtained from Dr. Adeline Hajjar (Department of Comparative Medicine, UW) and bred in-house as needed. Ascending infection studies were performed as previously described by us (23, 55). Briefly, female mice were individually paired with isogenic males for 2 days, then separated and monitored for 14 days for observable weight gain and palpation for the presence of pups. On day E15 of pregnancy, mice were anesthetized using 3% isoflurane, and 10 µL (~10^8^ CFU) of inoculum or sterile PBS was administered into the vaginal tract using a micropipette. Mice were left inverted for five additional minutes under anesthesia, then returned to their cages, and monitored until ambulation. Mice were monitored twice daily up to 3 days post-inoculation for signs of preterm birth (vaginal bleeding and/or pups in cage). At 3 days post-infection or earlier if preterm birth was observed, mothers were euthanized, and a mid-line laparotomy was performed to identify fetal injury and loss of pregnancy and to collect maternal and fetal tissues that included tissues of the lower genital tract (LGT), uterus, proximal and distal pups (whole pups), and proximal and distal placentas. Excised tissues were homogenized, serially diluted, and plated on TSB to determine the number of CFUs associated with maternal or fetal tissues. All data were normalized to total tissue weight. Homogenized tissue was then incubated overnight at 4°C in lysis buffer (0.15 M NH_4_Cl, 1 mM NaHCO_3_, pH 7.2) containing a complete protease inhibitor cocktail and pelleted, and supernatants were collected for further analysis as described below.

### Antibody administration

Pregnant WT mice (gestational age 12–13 days) were injected intraperitoneally with 100 µg of NA/LE (no azide, low endotoxin), anti-IL-10R mAb (Clone 1B1.3a, Cat No: 550012 BD Biosciences), or NA/LE isotype control (rat IgG1 k, Cat No: 554682 BD Biosciences) either 24 h prior to GBS inoculation or 0–96 h prior to euthanasia. The antibody clones and manufacturer are listed in Table S1.

### Flow cytometry

Uterine tissue was cut into small pieces and incubated in 1-mL digestion buffer (200 CDU/mL collagenase 1a, 1 mg/mL hyaluronidase, 150 μg/mL DNase, 20 mM HEPES, 100 U/mL penicillin, 100 μg/mL streptomycin, and 10 mg/mL BSA) for 1 h at 37°C. Placenta and digested uterine tissues were passed through a 40 µM filter to obtain single-cell suspensions. Approximately 1 × 10^6^ cells were stained with surface antibodies specific for CD11b, CD11c, F4/80, and Gr1 for 15 min. Cells were washed, fixed, and permeabilized using eBioscience Intracellular Fixation and Permeabilization Buffer Set prior to intracellular staining with antibodies specific for IL-10 for 30 min. Samples were acquired on an LSR-II flow cytometer, and analysis was performed using FlowJo version 10.7.1. Single-stained and fluorescence minus one controls were included. The antibody clones, respective fluorophore and manufacturer are listed in Table S1.

### Luminex and ELISA assays of murine tissues and cells

Tissue lysates from the mouse ascending infection model (above) were thawed and centrifuged at 10,000 × *g* for 5 min at 4°C to remove cell debris. Approximately 25 µL of the supernatants was used for cytokine analysis (IL-10, IL-1β, IL-6, GROα, TNF-α, MIP-2, MIP-1β, IFN-γ, IL-28, and IL-12p70) by Luminex assay (Procartaplex Multiplex Immunoassay, eBioscience) following the manufacturer’s instructions. Concentration values (pg/mL) were normalized to total tissue weight in grams.

Uterine macrophages were isolated from enzymatically digested pregnant mouse uterine tissue (gestational age: 13–14 days) using the EasySep Mouse F4/80 Positive Selection Kit (StemCell Technologies). Macrophages were allowed to adhere to 96-well plates overnight at a seeding density of 1 × 10^6^ /mL. Macrophages were then infected with GB37WT or GB37Δ*hylB* at an MOI of 1 for 4 h. Mock-infected PBS controls were also included. In some experiments, macrophages were pre-treated for an hour with 100 pg/mL rIL-10 (550070, BD) or control PBS. Supernatants were harvested, and cytokine concentrations were determined using a ProcartaPlex Luminex assay kit (ThermoFisher).

Bone marrow-derived macrophages (BMDM) were generated from cells isolated from femur and tibia bone marrow of WT, TLR2/4^−/−^, and IL-10^−/−^ mice. Briefly, cells were seeded on petri dishes at a density of 5 × 10^6^ /mL in 10 mL volume containing 10 ng/mL recombinant mouse M-CSF (Miltenyi) and incubated at 37°C, 5% CO_2_. After 3 days, an additional 10 mL media containing 10 ng/mL M-CSF was added. At day 7 of culture, media were aspirated, adherent BMDM were washed with ice-cold PBS, and cells were detached by incubating them with 0.05% Trypsin at 37°C for 30 min. Further detachment was achieved using disposable cell scrapers. Harvested BMDM were resuspended at 5 × 10^5^ /mL and allowed to adhere to flat-bottomed 96-well plates overnight at 37°C, 5% CO_2_. BMDM were stimulated with PBS control or 5 µg/mL CpG (Invivogen) for 4 h at 37°C 5%CO_2_. Supernatants were harvested, and cytokine concentration was determined using ProcartaPlex Luminex assays (ThermoFisher).

### Statistical analysis

Kruskal-Wallis test followed by Dunn’s multiple comparison test or Tukey’s multiple comparison test following ANOVA or the Mann-Whitney test was used to estimate differences as appropriate, and *P* < 0.05 was considered significant. Statistics were performed using GraphPad Prism version 10.0.1 for MacOS or Windows, GraphPad Software, USA.

## SUPPLEMENTAL MATERIAL

Supplemental Figures and Tables mbio.02049-23-s0001.docx Figures S1 to S11 and Table S1.

ASM does not own the copyrights to Supplemental Material that may be linked to, or accessed through, an article. The authors have granted ASM a non-exclusive, world-wide license to publish the Supplemental Material files. Please contact the corresponding author directly for reuse.

## References

[1] Verani JR , McGee L , Schrag SJ , Division of Bacterial Diseases, National Center for Immunization and Respiratory Diseases, Centers for Disease Control and Prevention (CDC) . 2010. Prevention of perinatal group B streptococcal disease--revised guidelines from CDC, 2010. MMWR Recomm Rep 59:1–36. 21088663

[2] Seale AC , Bianchi-Jassir F , Russell NJ , Kohli-Lynch M , Tann CJ , Hall J , Madrid L , Blencowe H , Cousens S , Baker CJ , Bartlett L , Cutland C , Gravett MG , Heath PT , Ip M , Le Doare K , Madhi SA , Rubens CE , Saha SK , Schrag SJ , Sobanjo-Ter Meulen A , Vekemans J , Lawn JE . 2017. Estimates of the burden of group B streptococcal disease worldwide for pregnant women, stillbirths, and children. Clin Infect Dis 65:S200–S219. doi:10.1093/cid/cix664 29117332 PMC5849940

[3] Russell NJ , Seale AC , O’Driscoll M , O’Sullivan C , Bianchi-Jassir F , Gonzalez-Guarin J , Lawn JE , Baker CJ , Bartlett L , Cutland C , Gravett MG , Heath PT , Le Doare K , Madhi SA , Rubens CE , Schrag S , Sobanjo-Ter Meulen A , Vekemans J , Saha SK , Ip M , GBS Maternal Colonization Investigator Group . 2017. Maternal colonization with group B Streptococcus and serotype distribution worldwide: systematic review and meta-analyses. Clin Infect Dis 65:S100–S111. doi:10.1093/cid/cix658 29117327 PMC5848259

[4] Seale AC , Hutchison C , Fernandes S , Stoesser N , Kelly H , Lowe B , Turner P , Hanson K , Chandler CIR , Goodman C , Stabler RA , Scott JAG . 2017. Supporting surveillance capacity for antimicrobial resistance: laboratory capacity strengthening for drug resistant infections in low and middle income countries. Wellcome Open Res 2:91. doi:10.12688/wellcomeopenres.12523.1 29181453 PMC5686477

[5] Stern R , Asari AA , Sugahara KN . 2006. Hyaluronan fragments: an information-rich system. Eur J Cell Biol 85:699–715. doi:10.1016/j.ejcb.2006.05.009 16822580

[6] Gouëffic Y , Guilluy C , Guérin P , Patra P , Pacaud P , Loirand G . 2006. Hyaluronan induces vascular smooth muscle cell migration through RHAMM-mediated PI3K-dependent Rac activation. Cardiovasc Res 72:339–348. doi:10.1016/j.cardiores.2006.07.017 16934786

[7] Taylor KR , Yamasaki K , Radek KA , Nardo AD , Goodarzi H , Golenbock D , Beutler B , Gallo RL . 2007. Recognition of hyaluronan released in sterile injury involves a unique receptor complex dependent on Toll-like receptor 4, CD44, and MD-2. J Biol Chem 282:18265–18275. doi:10.1074/jbc.M606352200 17400552

[8] Schledzewski K , Falkowski M , Moldenhauer G , Metharom P , Kzhyshkowska J , Ganss R , Demory A , Falkowska-Hansen B , Kurzen H , Ugurel S , Geginat G , Arnold B , Goerdt S . 2006. Lymphatic endothelium-specific hyaluronan receptor LYVE-1 is expressed by stabilin-1+, F4/80+, CD11b+ macrophages in malignant tumours and wound healing tissue in vivo and in bone marrow cultures in vitro: implications for the assessment of lymphangiogenesis. J Pathol 209:67–77. doi:10.1002/path.1942 16482496

[9] Yamawaki H , Hirohata S , Miyoshi T , Takahashi K , Ogawa H , Shinohata R , Demircan K , Kusachi S , Yamamoto K , Ninomiya Y . 2009. Hyaluronan receptors involved in cytokine induction in monocytes. Glycobiology 19:83–92. doi:10.1093/glycob/cwn109 18854367

[10] Francisco S , Billod J-M , Merino J , Punzón C , Gallego A , Arranz A , Martin-Santamaria S , Fresno M . 2021. Induction of TLR4/TLR2 interaction and heterodimer formation by low endotoxic atypical LPS. Front Immunol 12:748303. doi:10.3389/fimmu.2021.748303 35140704 PMC8818788

[11] Litwiniuk M , Krejner A , Speyrer MS , Gauto AR , Grzela T . 2016. Hyaluronic acid in inflammation and tissue regeneration. Wounds 28:78–88. 26978861

[12] Jiang D , Liang J , Fan J , Yu S , Chen S , Luo Y , Prestwich GD , Mascarenhas MM , Garg HG , Quinn DA , Homer RJ , Goldstein DR , Bucala R , Lee PJ , Medzhitov R , Noble PW . 2005. Regulation of lung injury and repair by toll-like receptors and hyaluronan. Nat Med 11:1173–1179. doi:10.1038/nm1315 16244651

[13] Puré E , Cuff CA . 2001. A crucial role for CD44 in inflammation. Trends Mol Med 7:213–221. doi:10.1016/s1471-4914(01)01963-3 11325633

[14] Jiang D , Liang J , Noble PW . 2011. Hyaluronan as an immune regulator in human diseases. Physiol Rev 91:221–264. doi:10.1152/physrev.00052.2009 21248167 PMC3051404

[15] DeGrendele HC , Estess P , Picker LJ , Siegelman MH . 1996. CD44 and its ligand hyaluronate mediate rolling under physiologic flow: a novel lymphocyte-endothelial cell primary adhesion pathway. J Exp Med 183:1119–1130. doi:10.1084/jem.183.3.1119 8642254 PMC2192320

[16] Stoop R , Gál I , Glant TT , McNeish JD , Mikecz K . 2002. Trafficking of CD44-deficient murine lymphocytes under normal and inflammatory conditions. Eur J Immunol 32:2532–2542. doi:10.1002/1521-4141(200209)32:9<2532::AID-IMMU2532>3.0.CO;2-A 12207337

[17] Alstergren P , Zhu B , Glogauer M , Mak TW , Ellen RP , Sodek J . 2004. Polarization and directed migration of murine neutrophils is dependent on cell surface expression of CD44. Cell Immunol 231:146–157. doi:10.1016/j.cellimm.2005.01.007 15919379

[18] Pritchard DG , Lin B , Willingham TR , Baker JR . 1994. Characterization of the group B streptococcal hyaluronate lyase. Arch Biochem Biophys 315:431–437. doi:10.1006/abbi.1994.1521 7986088

[19] Hynes WL , Walton SL . 2000. Hyaluronidases of Gram-positive bacteria. FEMS Microbiol Lett 183:201–207. doi:10.1111/j.1574-6968.2000.tb08958.x 10675584

[20] Wang Z , Guo C , Xu Y , Liu G , Lu C , Liu Y . 2014. Two novel functions of hyaluronidase from Streptococcus agalactiae are enhanced intracellular survival and inhibition of proinflammatory cytokine expression. Infect Immun 82:2615–2625. doi:10.1128/IAI.00022-14 24711564 PMC4019169

[21] Gochnauer TA , Wilson JB . 1951. Hyaluronidase production in vitro by streptococci isolated from bovine mastitis cases. Am J Vet Res 12:20–22. 14799725

[22] Kolar SL , Kyme P , Tseng CW , Soliman A , Kaplan A , Liang J , Nizet V , Jiang D , Murali R , Arditi M , Underhill DM , Liu GY . 2015. Group B Streptococcus evades host immunity by degrading hyaluronan. Cell Host Microbe 18:694–704. doi:10.1016/j.chom.2015.11.001 26651945 PMC4683412

[23] Vornhagen J , Quach P , Boldenow E , Merillat S , Whidbey C , Ngo LY , Adams Waldorf KM , Rajagopal L , Bello MGD . 2016. Bacterial hyaluronidase promotes ascending GBS infection and preterm birth. mBio 7:e00781-16. doi:10.1128/mBio.00781-16 27353757 PMC4937215

[24] Gendrin C , Vornhagen J , Armistead B , Singh P , Whidbey C , Merillat S , Knupp D , Parker R , Rogers LM , Quach P , Iyer LM , Aravind L , Manning SD , Aronoff DM , Rajagopal L . 2018. A nonhemolytic group B streptococcus strain exhibits hypervirulence. J Infect Dis 217:983–987. doi:10.1093/infdis/jix646 29244079 PMC5853813

[25] Coleman M , Armistead B , Orvis A , Quach P , Brokaw A , Gendrin C , Sharma K , Ogle J , Merillat S , Dacanay M , Wu T-Y , Munson J , Baldessari A , Vornhagen J , Furuta A , Nguyen S , Adams Waldorf KM , Rajagopal L , Coyne CB . 2021. Hyaluronidase impairs neutrophil function and promotes group B Streptococcus invasion and preterm labor in nonhuman primates. mBio 12:e03115-20. doi:10.1128/mBio.03115-20 33402537 PMC8545101

[26] Akgul Y , Word RA , Ensign LM , Yamaguchi Y , Lydon J , Hanes J , Mahendroo M . 2014. Hyaluronan in cervical epithelia protects against infection-mediated preterm birth. J Clin Invest 124:5481–5489. doi:10.1172/JCI78765 25384213 PMC4348952

[27] Pioli PA , Amiel E , Schaefer TM , Connolly JE , Wira CR , Guyre PM . 2004. Differential expression of toll-like receptors 2 and 4 in tissues of the human female reproductive tract. Infect Immun 72:5799–5806. doi:10.1128/IAI.72.10.5799-5806.2004 15385480 PMC517561

[28] Gonzalez JM , Xu H , Ofori E , Elovitz MA . 2007. Toll-like receptors in the uterus, cervix, and placenta: is pregnancy an immunosuppressed state? Am J Obstet Gynecol 197:296. doi:10.1016/j.ajog.2007.06.021 17826427

[29] Too LK , Yau B , Baxter AG , McGregor IS , Hunt NH . 2019. Double deficiency of toll-like receptors 2 and 4 alters long-term neurological sequelae in mice cured of pneumococcal meningitis. Sci Rep 9:16189. doi:10.1038/s41598-019-52212-7 31700009 PMC6838097

[30] Bebien M , Hensler ME , Davanture S , Hsu L-C , Karin M , Park JM , Alexopoulou L , Liu GY , Nizet V , Lawrence T . 2012. The pore-forming toxin beta hemolysin/cytolysin triggers p38 MAPK-dependent IL-10 production in macrophages and inhibits innate immunity. PLoS Pathog 8:e1002812. doi:10.1371/journal.ppat.1002812 22829768 PMC3400567

[31] Madureira P , Baptista M , Vieira M , Magalhães V , Camelo A , Oliveira L , Ribeiro A , Tavares D , Trieu-Cuot P , Vilanova M , Ferreira P . 2007. Streptococcus agalactiae GAPDH is a virulence-associated immunomodulatory protein. J Immunol 178:1379–1387. doi:10.4049/jimmunol.178.3.1379 17237385

[32] Madureira P , Andrade EB , Gama B , Oliveira L , Moreira S , Ribeiro A , Correia-Neves M , Trieu-Cuot P , Vilanova M , Ferreira P . 2011. Inhibition of IL-10 production by maternal antibodies against Group B streptococcus GAPDH confers immunity to offspring by favoring neutrophil recruitment. PLoS Pathog 7:e1002363. doi:10.1371/journal.ppat.1002363 22114550 PMC3219712

[33] Andrade EB , Alves J , Madureira P , Oliveira L , Ribeiro A , Cordeiro-da-Silva A , Correia-Neves M , Trieu-Cuot P , Ferreira P . 2013. TLR2-induced IL-10 production impairs neutrophil recruitment to infected tissues during neonatal bacterial sepsis. J Immunol 191:4759–4768. doi:10.4049/jimmunol.1301752 24078699

[34] Berkman N , John M , Roesems G , Jose PJ , Barnes PJ , Chung KF . 1995. Inhibition of macrophage inflammatory protein-1 alpha expression by IL-10. differential sensitivities in human blood monocytes and alveolar macrophages. J Immunol 155:4412–4418. 7594602

[35] Sun L , Guo R-F , Newstead MW , Standiford TJ , Macariola DR , Shanley TP . 2009. Effect of IL-10 on neutrophil recruitment and survival after Pseudomonas aeruginosa challenge. Am J Respir Cell Mol Biol 41:76–84. doi:10.1165/rcmb.2008-0202OC 19097982 PMC2701962

[36] Redford PS , Murray PJ , O’Garra A . 2011. The role of IL-10 in immune regulation during M. tuberculosis infection. Mucosal Immunol 4:261–270. doi:10.1038/mi.2011.7 21451501

[37] Ring S , Eggers L , Behrends J , Wutkowski A , Schwudke D , Kröger A , Hierweger AM , Hölscher C , Gabriel G , Schneider BE . 2019. Blocking IL-10 receptor signaling ameliorates Mycobacterium tuberculosis infection during influenza-induced exacerbation. JCI Insight 5:e126533. doi:10.1172/jci.insight.126533 30998505 PMC6542649

[38] Kamperschroer C , Goldstein R , Schneider PA , Kuang B , Eisenbraun MD . 2019. Utilization of lipopolysaccharide challenge in cynomolgus macaques to assess IL-10 receptor antagonism. J Immunotoxicol 16:164–172. doi:10.1080/1547691X.2019.1656683 31464151

[39] Peñaloza HF , Nieto PA , Muñoz-Durango N , Salazar-Echegarai FJ , Torres J , Parga MJ , Alvarez-Lobos M , Riedel CA , Kalergis AM , Bueno SM . 2015. Interleukin-10 plays a key role in the modulation of neutrophils recruitment and lung inflammation during infection by Streptococcus pneumoniae. Immunology 146:100–112. doi:10.1111/imm.12486 26032199 PMC4552505

[40] Gotsch F , Romero R , Kusanovic JP , Erez O , Espinoza J , Kim CJ , Vaisbuch E , Than NG , Mazaki-Tovi S , Chaiworapongsa T , Mazor M , Yoon BH , Edwin S , Gomez R , Mittal P , Hassan SS , Sharma S . 2008. The anti-inflammatory limb of the immune response in preterm labor, intra-amniotic infection/inflammation, and spontaneous parturition at term: a role for interleukin-10. J Matern Fetal Neonatal Med 21:529–547. doi:10.1080/14767050802127349 18609361 PMC6333088

[41] Gomez-Lopez N , Garcia-Flores V , Chin PY , Groome HM , Bijland MT , Diener KR , Romero R , Robertson SA . 2021. Macrophages exert homeostatic actions in pregnancy to protect against preterm birth and fetal inflammatory injury. JCI Insight 6:e146089. doi:10.1172/jci.insight.146089 34622802 PMC8525593

[42] Terrone DA , Rinehart BK , Granger JP , Barrilleaux PS , Martin JN , Bennett WA . 2001. Interleukin-10 administration and bacterial endotoxin-induced preterm birth in a rat model. Obstet Gynecol 98:476–480. doi:10.1016/s0029-7844(01)01424-7 11530133

[43] Robertson SA , Skinner RJ , Care AS . 2006. Essential role for IL-10 in resistance to lipopolysaccharide-induced preterm labor in mice. J Immunol 177:4888–4896. doi:10.4049/jimmunol.177.7.4888 16982931

[44] Sundaram S , Ashby CR , Pekson R , Sampat V , Sitapara R , Mantell L , Chen C-H , Yen H , Abhichandani K , Munnangi S , Khadtare N , Stephani RA , Reznik SE . 2013. N,N-dimethylacetamide regulates the proinflammatory response associated with endotoxin and prevents preterm birth. Am J Pathol 183:422–430. doi:10.1016/j.ajpath.2013.05.006 23770347 PMC3730776

[45] Olgun NS , Hanna N , Reznik SE . 2015. BQ-123 prevents LPS-induced preterm birth in mice via the induction of uterine and placental IL-10. Toxicol Appl Pharmacol 282:275–284. doi:10.1016/j.taap.2014.09.008 25230003

[46] Cheng SB , Sharma S . 2015. Interleukin-10: a pleiotropic regulator in pregnancy. Am J Reprod Immunol 73:487–500. doi:10.1111/aji.12329 25269386 PMC4382460

[47] Saraiva M , O’Garra A . 2010. The regulation of IL-10 production by immune cells. Nat Rev Immunol 10:170–181. doi:10.1038/nri2711 20154735

[48] Lai Z , Kalkunte S , Sharma S . 2011. A critical role of interleukin-10 in modulating hypoxia-induced preeclampsia-like disease in mice. Hypertension 57:505–514. doi:10.1161/HYPERTENSIONAHA.110.163329 21263114 PMC3621110

[49] Tinsley JH , South S , Chiasson VL , Mitchell BM . 2010. Interleukin-10 reduces inflammation, endothelial dysfunction, and blood pressure in hypertensive pregnant rats. Am J Physiol Regul Integr Comp Physiol 298:R713–R719. doi:10.1152/ajpregu.00712.2009 20053959

[50] Murphy SP , Fast LD , Hanna NN , Sharma S . 2005. Uterine NK cells mediate inflammation-induced fetal demise in IL-10-null mice. J Immunol 175:4084–4090. doi:10.4049/jimmunol.175.6.4084 16148158

[51] Murphy SP , Hanna NN , Fast LD , Shaw SK , Berg G , Padbury JF , Romero R , Sharma S . 2009. Evidence for participation of uterine natural killer cells in the mechanisms responsible for spontaneous preterm labor and delivery. Am J Obstet Gynecol 200:308. doi:10.1016/j.ajog.2008.10.043 PMC3893044 19114277

[52] Davies HD , Adair C , McGeer A , Ma D , Robertson S , Mucenski M , Kowalsky L , Tyrell G , Baker CJ . 2001. Antibodies to capsular polysaccharides of group B Streptococcus in pregnant Canadian women: relationship to colonization status and infection in the neonate. J Infect Dis 184:285–291. doi:10.1086/322029 11443553

[53] Spaetgens R , DeBella K , Ma D , Robertson S , Mucenski M , Davies HD . 2002. Perinatal antibiotic usage and changes in colonization and resistance rates of group B streptococcus and other pathogens. Obstet Gynecol 100:525–533. doi:10.1016/s0029-7844(02)02068-9 12220773

[54] Skerrett SJ , Wilson CB , Liggitt HD , Hajjar AM . 2007. Redundant toll-like receptor signaling in the pulmonary host response to Pseudomonas aeruginosa. Am J Physiol Lung Cell Mol Physiol 292:L312–L322. doi:10.1152/ajplung.00250.2006 16936244

[55] Vornhagen J , Armistead B , Santana-Ufret V , Gendrin C , Merillat S , Coleman M , Quach P , Boldenow E , Alishetti V , Leonhard-Melief C , Ngo LY , Whidbey C , Doran KS , Curtis C , Waldorf KMA , Nance E , Rajagopal L . 2018. Group B streptococcus exploits vaginal epithelial exfoliation for ascending infection. J Clin Invest 128:1985–1999. doi:10.1172/JCI97043 29629904 PMC5919824

