# Supplementary material for: GBS hyaluronidase mediates immune suppression in a TLR2/4- and IL-10-dependent manner during pregnancy-associated infection
Source: mBio. 2023 Sep 25;14(5):e02049-23. doi: 10.1128/mbio.02049-23 (PMC10653848; doi:10.1128/mbio.02049-23)
Supplement: Supplemental Figures and Tables — Figures S1 to S11 and Table S1. [file mbio.02049-23-s0001.docx]

**Supplemental Figures**

**Figure S1:** **Ascending infection of GB37 is not significantly different between WT, TLR2 and TLR4 deficient mice.**

 Mice deficient for TLR2 (TLR2^-/-)^ and TLR4 (TLR4^-/-)^ were vaginally inoculated with approximately 1 x 10^8^ CFU of hyaluronidase proficient WT GBS (strain GB37) or isogenic hyaluronidase deficient strain GB37Δ*hylB* (*n* = 11-12/group). Data from WT and TLR2/4 (TLR2/4^-/-)^ deficient mice (shown in Fig 1) are also shown here for comparison to data from the single knock-out mice. Tissues of LGT, uterus, placentas, and pups were homogenized, and bacterial burden was enumerated by serial dilution and plating. Individual data with the median is shown for each tissue. Triangle symbols indicate mice that exhibited preterm labor. Kruskal-Wallis test with Dunn’s Multiple Comparison test was used to assess statistical significance between groups and significant differences are shown (** p < 0.01, **** p<0.0001).

**Figure S2: CFU were not recovered after vaginal inoculation of PBS in pregnant WT and TLR2/4 deficient mice**.

Wild type (WT) mice and mice deficient for TLR2 and TLR4 (TLR2/4^-/-)^ were vaginally inoculated with PBS (10μl, *n* = 5/group). At 72 hours post inoculation, tissues of LGT, uterus, placentas, and pups were homogenized, and bacterial burden was enumerated by serial dilution and plating on TSA and/or CHROMagar Strep B (DRG International Inc.). No bacteria were recovered from any tissues.

**Figure S3 – Flow cytometry gating strategy**.


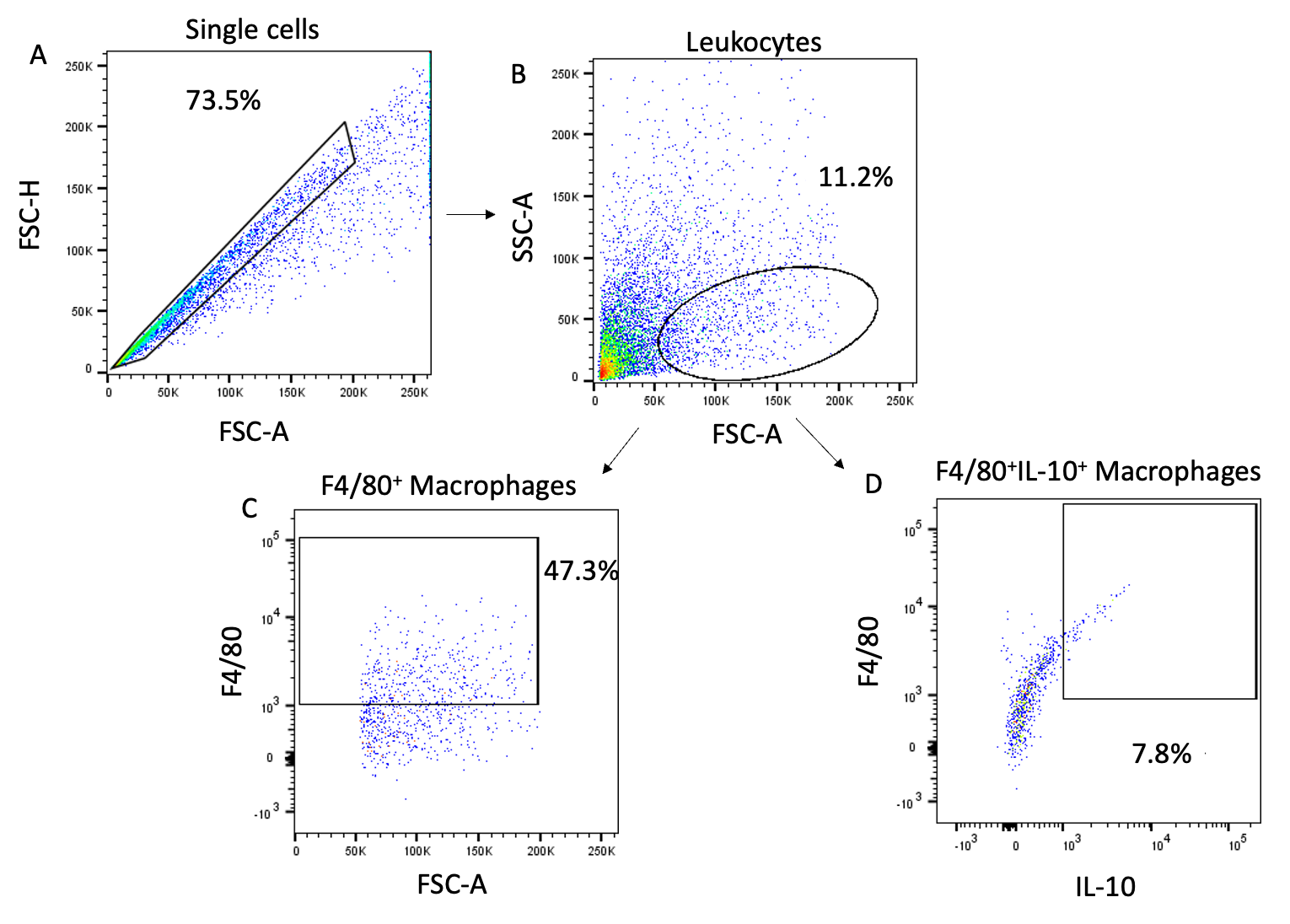
Representative dot plots of flow cytometry analysis of immune cells in mouse uterus and placenta. GB37 or GB37Δ*hylB*-infected tissues were first gated for single cells based on FSC-A vs FSC-H (A) and then leukocytes were broadly gated based on FSC-A vs SSC-A (B). This population was then gated for total F4/80^+^ macrophages (C) or double positive F4/80^+^IL-10^+^ macrophages (D).

**Figure S4: Representative dot plots showing the frequencies of F4/80^+^ macrophages in uterine tissues.**

Frequencies of F4/80^+^ macrophages in uterine tissues of pregnant WT, TLR2/4 deficient, and CD44 deficient mice inoculated with GB37 or GB37Δ*hylB* (*n* = 9-10/group) was determined by flow cytometry and is shown as percentages of the indicated population.


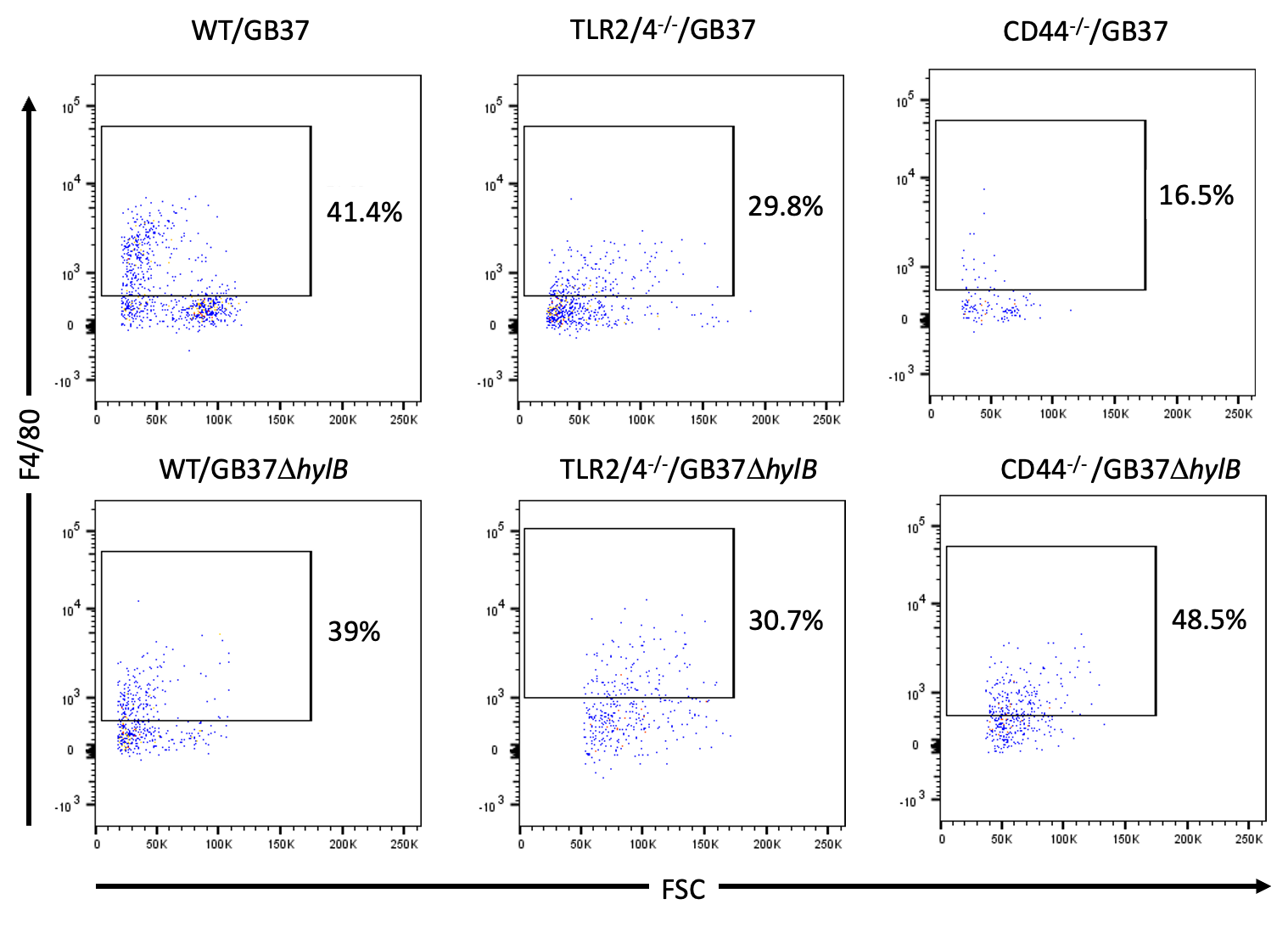


**Figure S5: Representative dot plots showing the frequencies of F4/80^+^ IL-10^+^** **macrophages in uterine tissues.**

Frequencies of F4/80^+^IL-10^+^ macrophages in uterine tissue from pregnant WT, TLR2/4 deficient, and CD44 deficient mice that were inoculated with GB37 or GB37Δ*hylB* (*n* = 9-10/group). Frequencies were determined by flow cytometry and are shown as percentages of the indicated population.


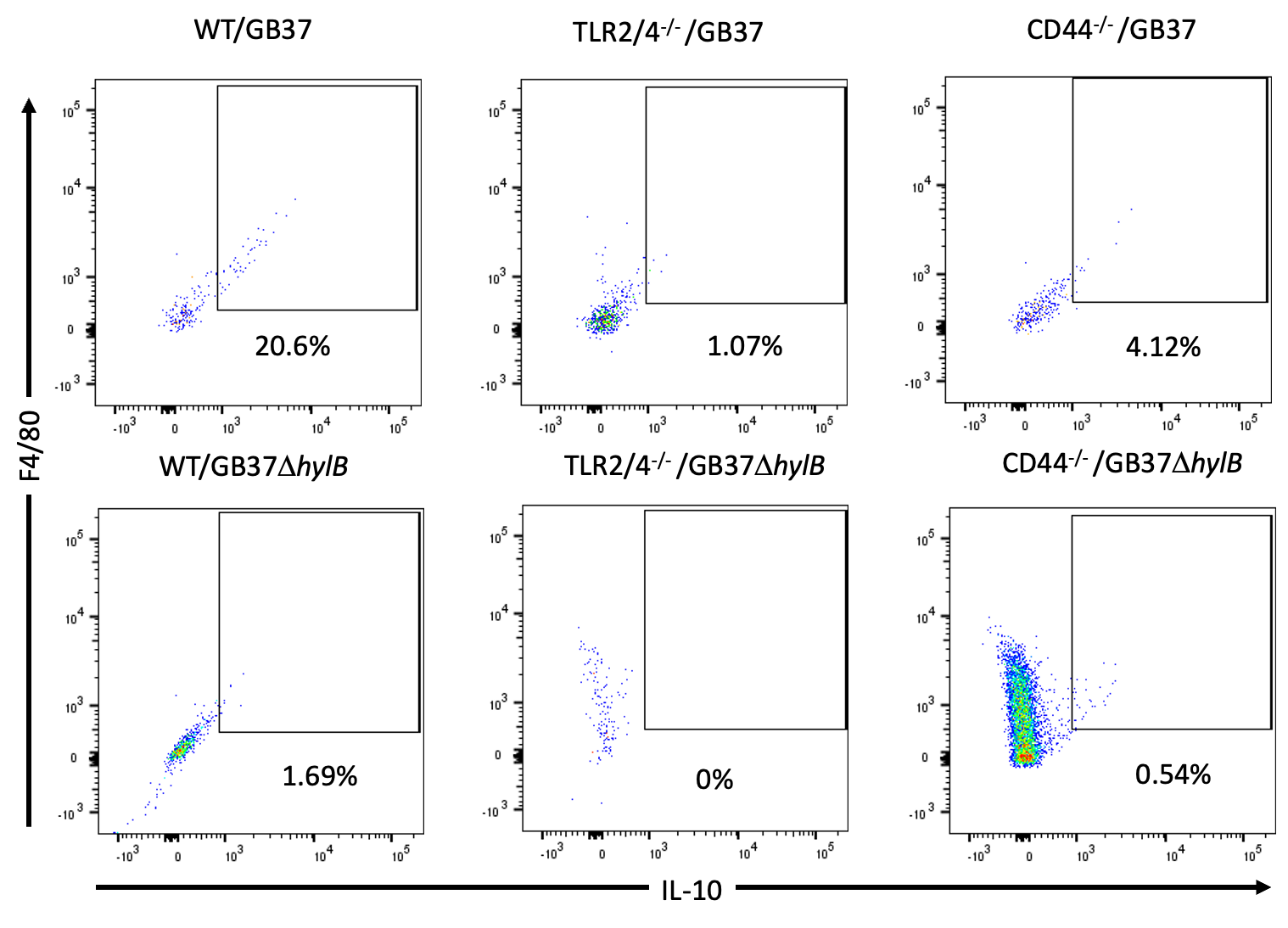


**Figure S6: Representative dot plots showing the frequencies of F4/80^+^ macrophages in placental tissues.**

The frequencies of F4/80^+^ macrophages in placental tissue from WT, TLR2/4 deficient, and CD44 deficient mice that were inoculated with GB37 or GB37Δ*hylB* (*n* = 9-10/group). Frequencies were determined by flow cytometry and are shown as percentages of the indicated population.


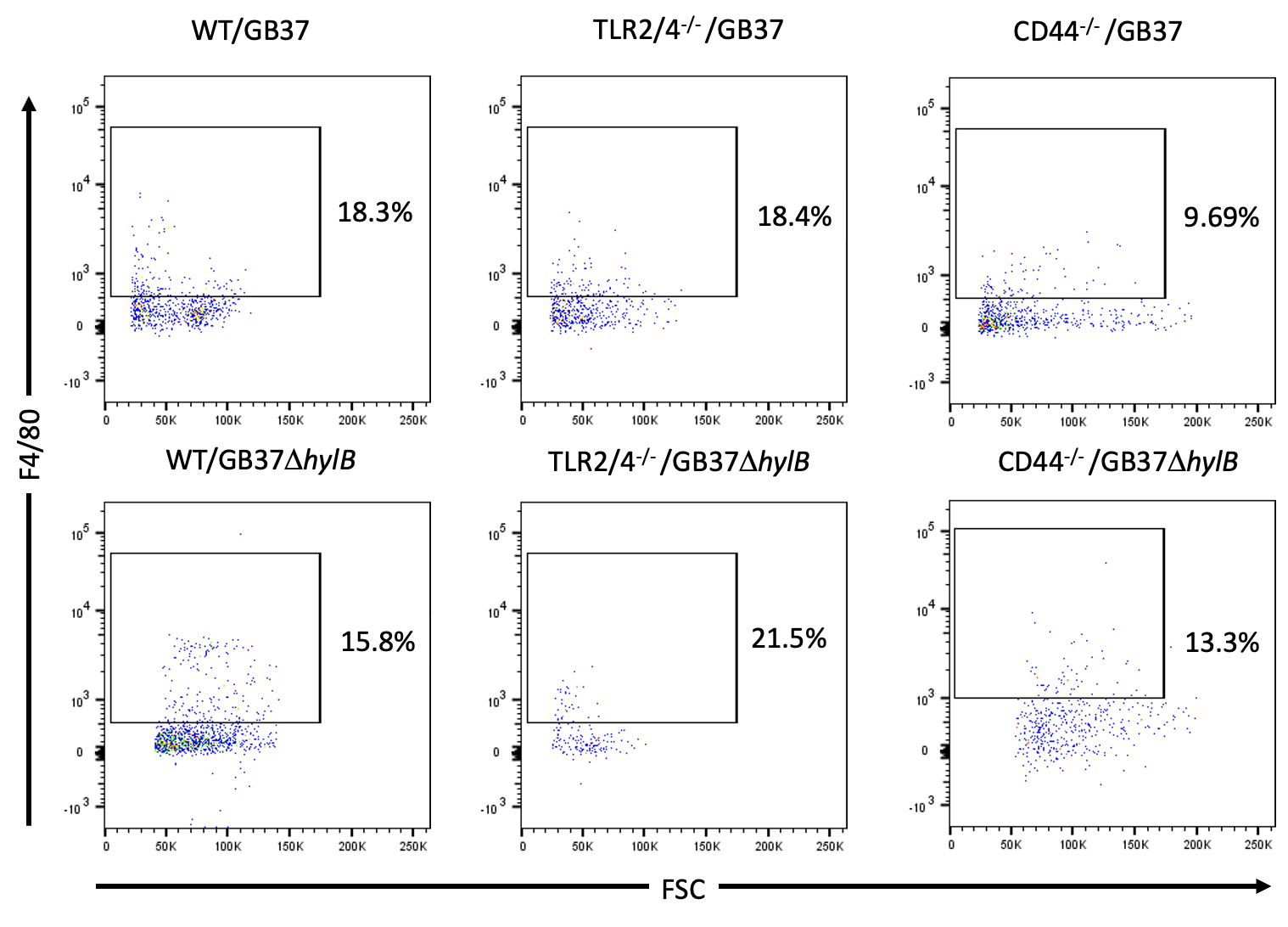


**Figure S7: Representative dot plots showing the frequencies of F4/80^+^** **IL-10^+^ macrophages in placental tissues.**

The frequencies of F4/80^+^ IL-10^+^ macrophages in placental tissue from WT, TLR2/4 deficient, and CD44 deficient mice that were inoculated with GB37 or GB37Δ*hylB* (*n* = 9-10/group). Frequencies were determined by flow cytometry and are shown as percentages of the indicated population.

**
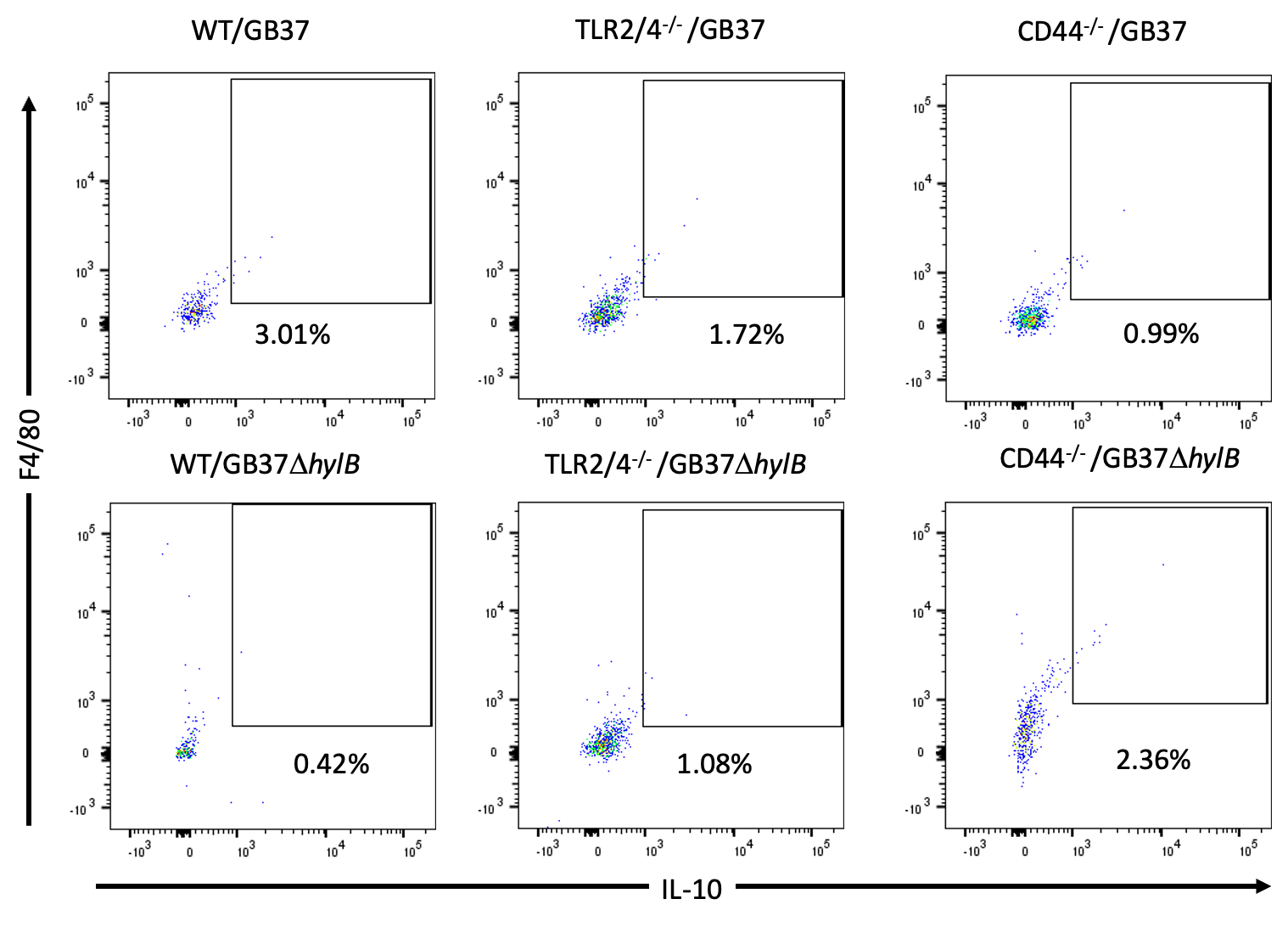
**

**Figure S8: Neutrophil recruitment in uterus and placental tissues of WT, TLR2/4 deficient, and CD44 deficient mice.**

Uterine and placental samples from WT, TLR2/4 deficient, and CD44 deficient mice infected with GB37 or GB37Δ*hylB* (*n* = 9-10/group) were processed into single-cell suspensions, stained, and analyzed by flow cytometry. Neutrophils (Gr1^+^) are shown as percentages of the total leukocytes for each tissue type. Triangle symbols indicate mice that exhibited preterm labor. Kruskal-Wallis test with Dunn’s Multiple Comparison test was used to assess statistical significance between groups, data are not significantly different.

**Figure S9: Flow cytometry of immune cells in PBS inoculated pregnant WT and TLR2/4 deficient mice.**

Uterine and placental samples, from pregnant WT and TLR2/4 deficient mice that were vaginally inoculated with PBS (*n* = 5/group), were processed into single-cell suspensions, stained, and analyzed by flow cytometry. Frequencies of F4/80^+^ macrophages and F4/80^+^ IL10^+^ macrophages are shown as percentages of the indicated population for each tissue type. Neutrophils (Gr1^+^) are shown as percentages of the total leukocytes for each tissue type. Mann-Whitney test was used to assess statistical significance between groups, data are not significantly different p > 0.05).

**Figure S10: WT and TLR2/4 deficient macrophages release IL-10 in response to CpG**

WT, TLR2/4^-/-^ or IL-10^-/-^ macrophages (5 x 10^5^ cells/ml) were unstimulated (blue) or stimulated with 5 μg/ml CpG (red) for 4 hours at 37^o^C, 5% CO_2_.

After 4 hours, IL-10 concentrations in the supernatant were quantified via Luminex. Statistical significance was calculated using 2-way ANOVA with Tukey’s multiple comparison test (** p <0.01).

**Figure S11: Administration of anti-IL-10 receptor antibody blocks IL-10R binding in uterine tissues in the duration relevant for GBS infection.**

Female WT mice received 100μg of NA/LE anti-IL-10R mAb intraperitoneally (Clone 1B1.3a 550012 BD), 0-96h prior to euthanasia. Control mice received isotype control NA/LE Rat IgG1 mAb (*n=3/group*). Single cell suspensions were generated from enzymatically digested uterine tissue and the frequency of IL-10R expression on uterine cells was determined by flow cytometry using rat anti-mouse IL-10R conjugated with PE (Clone 1B1.3a, 559914 BD). Administration of the anti-IL-10R mAb resulted in reduced detection of IL-10R^+^ cells in the uterus up to 48 hrs. Data shows mean ± =SEM. * p < 0.05, 2-way ANOVA with Sidak’s multiple comparison test.

**Table S1:**

**Antibodies used in Flow Cytometry**

| **Antibody** | **Fluorophore** | **Clone** | **Manufacturer** |
| --- | --- | --- | --- |
| CD11b | APC | M1/70 | BD Biosciences |
| CD11c | PE | N418 | eBioscience |
| F4/80 | Brilliant Violet 510 | BM8 | Biolegend |
| Gr1 | AlexaFluor 700 | RB6-8C5 | Biolegend |
| IL-10 | FITC | JES5-16E3 | Invitrogen |
| IL-10R | PE | 1B1.3A | Biolegend |

**Antibodies used *in vivo***

| **Antibody** | **Clone** | **Manufacturer** |
| --- | --- | --- |
| IL-10R | 1B1.3A | Biolegend |
| Isotype control | R3-34 | BD Biosciences |
